# Selection of internal reference genes for SYBR green qRT-PCR studies of rhesus monkey (*Macaca mulatta*) tissues

**DOI:** 10.1186/1471-2199-9-78

**Published:** 2008-09-10

**Authors:** Kung Ahn, Jae-Won Huh, Sang-Je Park, Dae-Soo Kim, Hong-Seok Ha, Yun-Ji Kim, Ja-Rang Lee, Kyu-Tae Chang, Heui-Soo Kim

**Affiliations:** 1 Division of Biological Sciences, College of Natural Sciences, Pusan National University, Busan, 609-735, Republic of Korea; 2 National Primate Research Center (NPRC), KRIBB, Ochang, Chungbuk, 363-883, Republic of Korea; 3 PBBRC, Interdisciplinary Research Program of Bioinformatics, College of Natural Sciences, Pusan National University, Busan, 609-735, Republic of Korea

## Abstract

**Background:**

The rhesus monkey (*Macaca mulatta*) is a valuable and widely used model animal for biomedical research. However, quantitative analyses of rhesus gene expression profiles under diverse experimental conditions are limited by a shortage of suitable internal controls for the normalization of mRNA levels. In this study, we used a systematic approach for the selection of potential reference genes in the rhesus monkey and compared their suitability to that of the corresponding genes in humans.

**Results:**

Eight housekeeping genes (HKGs) (*GAPDH, SDHA, ACTB, RPL13A, RPL32, UBA52, PGK1Y*, and *YWHAZ*) from rhesus monkeys and humans were selected to test for normalization of expression levels in six different tissue types (brain, colon, kidney, liver, lung, and stomach). Their stability and suitability as reference genes were validated by *geNorm*, *NormFinder *and *BestKeeper *programs. Intriguingly, *RPL13A *and *RPL32 *were selected as ideal reference genes only in rhesus monkeys.

**Conclusion:**

The results clearly indicated the necessity of using different reference genes for normalization of expression levels between rhesus monkeys and humans in various tissues.

## Background

The rhesus monkey (*Macaca mulatta*) has been one of the most valuable experimental model species for biomedical studies in various research fields including microbiology, neuroscience, and biochemistry. Recently, the genome sequence of the rhesus monkey was determined by the Rhesus Macaque Genome Sequencing and Analysis Consortium [1]. The consortium revealed that the average sequence similarity between humans and rhesus monkeys is 93%. Approximately twenty thousand genes were annotated or predicted by the human-based comparative analysis. In order to conduct biomedical research in areas such as infectious disease, cancer research, and drug development using various monkey tissues, quantitative gene expression analysis is essential.

Quantitative real-time PCR (qRT-PCR) is a recently developed and popularized molecular technique that can be used for gene expression studies that require highly sensitive and accurate quantification of mRNA levels from various samples [2]. These PCR-based analyses allow investigators to analyze a limited amount of material of interest while guaranteeing accurate quantification of target mRNAs. However, to achieve optimal results in qRT-PCR analysis, certain steps are indispensable including: (1) quality control of the material of interest and the primer sets, (2) determination of the PCR efficiency, and (3) selection of suitable internal control genes for normalization [3]. The availability of advanced biomaterials including sophisticated PCR instrumentation, kits for RNA isolation from various materials, and highly purified Taq polymerase, has overcome many of these difficult steps with the exception of identifying appropriate internal control genes [4]. Different expression profiling results from the same target genes can be obtained by using different HKGs as internal references [5]. Therefore, selection of proper HKGs is important for the accurate analysis and normalization of data generated by real-time qRT-PCR [2].

Before the systematic selection of reference genes, researchers interested in quantitative expression analysis have used empirically determined housekeeping genes such as *ACTB *and *GAPDH*. However, recent studies have provided solid evidence that transcription levels of housekeeping genes vary between cell types [6-11], developmental stages [12,13], and experimental conditions [14,15], individuals [16-20]. Therefore, stable reference gene selection procedures have to be undertaken before quantitative mRNA expression analysis can be conducted.

In this study, three different specific tools (*geNorm, Normfinder*, and *BestKeeper*) that can be used for the validation of the stability of selected HKGs (*GAPDH, SDHA, ACTB, RPL13A, RPL32, UBA52, PGK1Y*, and *YWHAZ*) were investigated in rhesus monkey and human tissues (brain, colon, kidney, liver, lung, stomach) using real-time qRT-PCR with SYBR green.

## Results and discussion

### Reference gene and qRT-PCR primer selection

For the selection of suitable reference genes from the human and rhesus monkey genomes, the eight most commonly used and available HKGs were selected: Hydroxymethylbilane synthase (*HMBS*), Beta-actin (*ACTB*), Glyceraldehyde-3-phosphate dehydrogenase (*GAPDH*), Ribosomal protein 13 (*RPL13A*), Ribosomal protein 32 (*RPL32*), Tyrosine 3-monooxygenase (*YWHAZ*), Hypoxanthine phosphoribosyltransferase 1(*HPRT1*), and TATA box-binding protein (*TBP*). Rhesus monkey HKGs are identified by comparison of orthologous loci of the human HKGs. Summarized sequence information for the human and rhesus monkey genes is shown in Table 1. This gene information was also used to design qRT-PCR primers (Table 2).

**Table 1:** GenBank accession numbers and functions of 8 reference genes in rhesus monkeys and humans

Symbol	Gene Name	Function	Accession Number	
			Human	Rhesus
			
GAPDH	Glyceraldehyde-3-phospate dehydrogenase	Glycolitic enzyme	NM_002046.3	XM_001105471.1
ACTB	Beta-actin	Cytoskeletal structural protein	NM_001101.2	NM_001033084.1
RPL13A	Ribosomal protein L13A	Member of ribosome protein	NM_012423.2	XM_001093017.1
RHL32	60S ribosomal protein L32	Member of ribosome protein	NM_001007074.1	XM_001117016.1
YWHAZ	Tyrosine 3-monooxygenase	Signal transduction by binding to phosphorilated serine residue on a variaty of signaling molecules	NM_145690.1	XM_001098275.1
HMBS	hydroxymethylbilane synthase	Third enzyme of the heme biosynthetic pathway and catalyzes the head to tail condensation of four porphobilinogen molecules into the linear hydroxymethylbilane.	NM_000190.3	XM_001101850.1
TBP	TATA box binding protein	Transcription initiation from RNA polymerase II promoter	NM_003194.3	XM_001085016.1
HPRT1	Hypoxanthine phosphoribosyltransferase 1	Metabolic salvage of purines in mammals	NM_000194.2	XM_001097691.1

**Table 2:** Details of primers used for quantitative RT-PCR

Gene	Forward Primer Sequence [5' → 3']	Position in cDNA	Reverse Primer Sequence [5' → 3']	Position in cDNA	Products Size
GAPDH	GAAATCCCATCACCATCTTCCAGG	4th Exon	GAGCCCCAGCCTTCTCCATG	5th Exon	120 bp
ACTB	CCTGGCACCCAGCACAAT	4th Exon	GGGCCGGACTCGTCATAC	5th Exon	144 bp
RPL13A	CCTGGAGGAGAAGAGGAAAGAGA	7th Exon	TTGAGGACCTCTGTGTATTTGTCAA	8th Exon	126 bp
RPL32	CAACATTGGTTATGGAAGCAACA	3th Exon	TGACGTTGTGGACCAGGAACT	3th Exon	80 bp
YWHAZ	TCCTTTGCTTGCATCCCAC	6th Exon	AAGGCAGACAATGACAGACCA	6th Exon	132 bp
HMBS	ACCAAGGAGCTTGAACATGC	5th Exon	GAAAGACAACAGCATCATGAG	7th Exon	145 bp
TBP	TGCACAGGAGCCAAGAGTGAA	5, 6th Exon	CACATCACAGCTCCCCACCA	6th Exon	132 bp
HPRT1	AATTATGGACAGGACTGAACGTCTTGCT	2, 3th Exon	TCCAGCAGGTCAGCAAAGAATTTATAGC	3th Exon	117 bp

### Amplification efficiency

The expression levels of HKGs were analyzed using the relative quantification (delta-Ct method) [21]. The amplification efficiencies and correlation coefficients (R^2^) of the eight HKGs were generated using the slopes of the standard curves obtained by serial dilutions. Standard curves with a ten-fold dilution series were used to calculate the amplification efficiency (additional files 1 and 2). Similar amplification efficiencies for the corresponding human and rhesus monkey reference genes are a prerequisite for accurate real-time RT-PCR quantification. The amplification efficiency was calculated by the formula: efficiency (%) = (10^(-1/slope) ^-1)*100. The efficiency range of the qRT-PCR amplifications for all of the genes tested was between 80% and 115%. Overall, similar efficiency levels were observed between human and rhesus samples (additional file 3). The amplification specificity for each qRT-PCR analysis was confirmed by melting curve analysis (additional files 4 and 5).

### Data analysis

We analyzed eight housekeeping genes for the selection of stable HKGs using three authorized programs (*geNORM, NormFinder*, and *BestKeeper*) [22-24].

#### geNorm

The normalization step must be conducted very carefully for accurate gene expression level analysis. However, the selection of stable HKGs for normalization is very complicated. *geNorm *is a useful tool for the selection of the most stable internal control genes using the principle that the expression ratio of two ideal internal control genes is identical in all tested samples [22].

**Figure 1:**
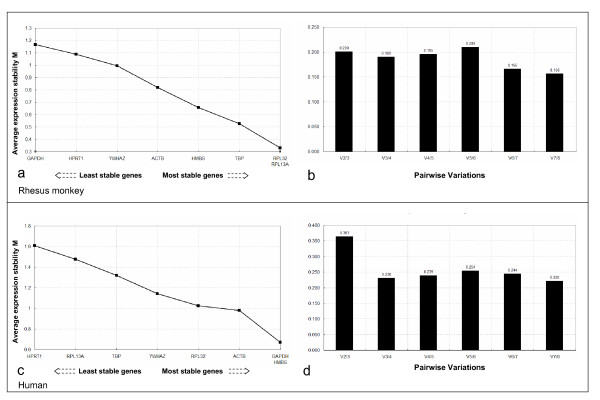
**Selection of the most suitable reference genes for normalization in rhesus monkey (a and b) and human (c and d) samples using *geNorm *analysis**. Stepwise exclusion of the least stable genes by calculating the average expression stability (a and c) was conducted. The value of M was calculated for each gene, and the least stable gene with the highest M value was automatically excluded for the next round of calculations. The x-axis from left to right indicates the ranking of the genes according to their expression stability. Determination of the optimal number of reference genes for normalization (b and d) was conducted. The software calculates the normalization factor from at least two genes at which the variable V defines the pair-wise variation between two sequential normalization factors.

Using the *geNorm *program, the eight HKGs selected for normalization were ranked according to their M values. The M value is the average pair-wise variation of a particular gene with all other control genes. The M values for *RPL13A, RPL32, TBP, HMBS, ACTB, YWHAZ, HPRT1*, and *GAPDH *in rhesus were lower than 1.5.*RPL32 *and *RPL13A *displayed the most stability of all eight genes according to *geNorm *analysis. Both genes encode components of the 60S ribosomal subunit that is involved in protein biosynthesis (Fig. 1a). In contrast to rhesus monkeys, human tissue analysis indicated that *GAPDH *and *HMBS *were the most stable genes (Fig. 1c). The M values of all tested human genes except for *HPRT1 *were lower than 1.5. To investigate the optimal number of reference genes, it was determined whether the stepwise inclusion of less stable genes significantly affected the normalization factors, considering the term V (Fig. 1).

As the most stable reference genes, *RPL32 *and *RPL13A *yielded a V value of 0.200 in the rhesus monkey (Fig. 1b). That value indicated that it would not be necessary to include additional reference genes in qRT-PCR of rhesus monkey tissues. However, in the case of humans, the addition of a third and fourth gene significantly affected the reliability of the selected HKGs (Fig. 1d).

The results of the *geNorm *tool indicated different results between humans and rhesus monkeys. *RPL32 *and *RPL13A *in the rhesus monkey and *GAPDH, HMBS, ACTB *and *RPL32 *in humans are the most stable HKGs for the accurate calculation of normalization factors.

#### NormFinder

*NormFinder *is another reference tool for identifying the ideal normalization genes among various candidate HKGs [23]. *NormFinder *ranks the various candidate reference genes according to their expression variation between inter and intra groups. This analysis identified *HMBS *and *RPL32 *as the most stable genes in rhesus monkeys and humans, respectively (Table 3). These rankings appear to be generally different from the results of *geNorm*. However, *RPL32*, *GAPDH *and *ACTB *in humans and *HMBS *and *RPL32 *in rhesus monkeys are still ranked as the most stable reference genes. Although *geNorm *and *NormFinder *did not identify the exact same reference genes, *GAPDH *in humans and *HMBS *in rhesus monkeys are still considered candidate reference genes.

**Table 3:** Candidate reference genes for normalization and the best combination of two genes listed according to their expression stability calculated by the *NormFinder *program

Ranking order	Gene name	Stablility value of rhesus monkey	Gene name	Stablility value of human
1	HMBS	0.349	RPL32	0.39
2	RPL32	0.4	GAPDH	0.594
3	RPL13A	0.434	ACTB	0.668
4	ACTB	0.589	TBP	0.705
5	HPRT1	0.649	YWHAZ	0.754
6	TBP	0.687	HMBS	0.936
7	YWHAZ	0.709	RPL13A	1.073
8	GAPDH	0.837	HPRT1	1.185

**Figure 2:**
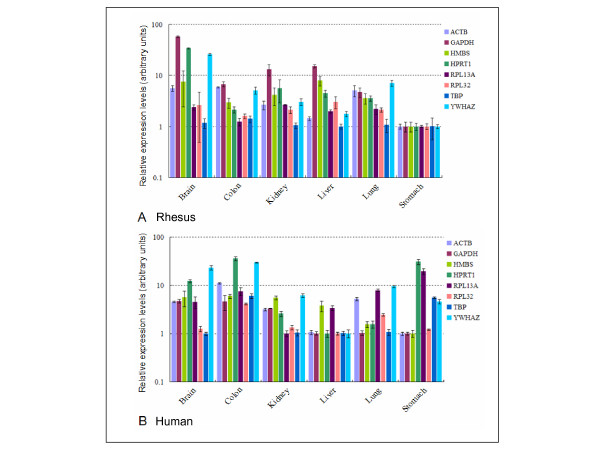
**Logarithmic histogram of the expression levels of 8 internal reference genes determined in 6 different tissues**. Relative expression levels of rhesus monkey (A) and human (B) with six different tissues. The respective bar and genes name are: actin beta (ACTB: light blue bar), Glyceraldehyde-3-phospate dehydrogenase (GAPDH: light purple bar), Hydroxymethylbilane synthase (HMBS: light green bar), Hypoxanthine phosphoribosyltransferas 1 (HPRT1: green bar) Ribosomal protein L13A (RPL13A: dark purple bar), 60S ribosomal protein L32 (RPL32: pink bar), TATA box binding protein (TBP: dark blue bar), Tyrosine 3-monooxygenase (YWHAZ: sky blue bar).

Intriguingly, human *HMBS *showed opposite results between *geNorm *and *Normfinder *program. In the *geNorm *software, the most important factor is gene expression stability (M). The principle of *geNorm *is that the expression ratio of two ideal internal control genes is identical in all tested samples [22]. Thus, the *geNorm *program did not consider the other HKGs. However, *Normfinder *program focuses on the two genes with the least intra-and inter-group expression variation. In other words, the *Normfinder *program considered other HKGs for the selection of good HKGs. For example, high rate of expression variation of *HMBS *among HKGs could result in the discordance between *Normfinder *and *geNorm *(Fig. 2).

#### BestKeeper

The BestKeeper program index was created using the geometric mean of each candidate gene's Ct values [24]. Initial analysis of the data, based on the inspection of raw Ct values, calculated variations for all the housekeeping genes in the six samples (SD [± Ct] and CV [%Ct]), and showed an overall stability in gene expression. Genes with SD values higher than 1 were considered inconsistent. *ACTB, HMBS, RPL13A, RPL32, TBP, YWHAZ *showed a SD value lower than 1 in the rhesus monkey. Among them, *RPL13A *(SD = 0.22) had the lowest Ct value variation. *GAPDH *had the highest Ct value variation. In human, *HMBS *and *RPL32 *exhibit SD values lower than 1. *RPL32 *had the lowest Ct value variation and *HPRT1 *had the highest Ct value variation (Table 4). In the case of *HMBS *and *RPL32*, *BestKeeper *also showed a different result compared to *Normfinder *and *geNorm *program, respectively. Because the variation of Ct value of *HMBS *and *RPL32 *is ranked very low, the *BestKeeper *program indicated *HMBS *and *RPL32 *as good candidate HKGs. However, compared to BestKeeper, geNorm is considered a pair-wise variation between the gens as the most important factor. Thus *geNorm *did not indicate one gene as a best reference gene but indicated a gene pair.

**Table 4:** Results from Bestkeeper descriptive statistical analysis

	GAPDH		ACTB		RPL13A		RPL32		YWHAZ		HMBS		TBP		HPRT1	
n	6		6		6		6		6		6		6		6	

	human	rhesus monkey	human	rhesus monkey	human	rhesus monkey	human	rhesus monkey	human	rhesus monkey	human	rhesus monkey	human	rhesus monkey	human	rhesus monkey

geo Mean [CP]	17.01	15.79	15.03	16.31	19.31	16.83	22.43	25.36	19.30	20.71	27.06	29.87	19.65	17.53	22.89	23.47
ar Mean [CP]	17.04	15.86	15.08	16.31	19.36	16.83	22.44	25.37	19.34	20.72	27.08	29.88	19.71	17.54	22.98	23.50
min [CP]	15.84	13.97	13.25	15.75	17.43	16.53	21.12	24.44	17.62	19.87	26.19	28.26	17.74	17.10	20.44	21.87
max [CP]	18.08	17.85	16.65	16.92	21.71	17.28	23.16	25.81	20.22	21.42	28.76	30.95	22.63	18.15	25.61	24.71
std dev [± CP]	1.02	1.34	1.04	0.30	1.04	0.22	0.63	0.35	1.10	0.54	0.91	0.72	1.31	0.30	1.95	1.04
CV [% CP]	6.00	8.45	6.92	1.85	5.37	1.33	2.79	1.39	5.71	2.60	3.35	2.41	6.62	1.73	8.49	4.41

Based on the Bestkeeper program, the *RPL13A *gene in the rhesus monkey and the RPL32 gene in the human were selected as the best reference genes.

### Validation

To validate our results from the rhesus monkey, one unknown rhesus monkey was tested using same reference genes. And results were analyzed for the selection of stable HKGs using three authorized programs (*geNORM, NormFinder*, and *BestKeeper*) (additional files 6, 7 and 8). Although, slight differences were observed, overall patterns were coincided with our results of *RPL13A *and *RPL32 *as ideal reference genes in rhesus monkeys.

## Conclusion

In this study, we investigated for the first time the most reliable HKGs for the normalization of real-time qRT-PCR data in rhesus monkey tissues (brain, colon, kidney, liver, lung, stomach) using three programs (*geNorm, Normfinder, and Bestkeeper*). The results from the rhesus monkey were compared to those of humans.

Our results indicated that the two most stable genes, *RPL13A *and *RPL32*, covering a broad expression range, could be used as reference genes for relative gene quantification and normalization purposes in gene profiling studies of the rhesus monkey as determined by three reference gene identification programs.

In humans, *RPL32, HMBS *and *GAPDH *were found to be suitable reference genes. In the case of *RPL32*, two programs (*Bestkeepe*r and *Normfinder*) identified this gene as a reliable reference gene. However, *geNorm *indicated that *RPL32 *was an unsuitable reference gene by virtue of having a high M value. Likewise, the *GAPDH *gene was rejected by the Bestkeeper program as a result of having a high SD [± Ct] value. Finally, *HMBS *was ranked as the sixth best gene by the *Normfinder *program.

Intriguingly, the *GAPDH *and *ACTB *genes are the most widely used reference genes in human studies. However, *GAPDH *and *ACTB *are ranked as the least stable genes in the rhesus monkey. Genomic and environmental differences between humans and rhesus monkeys might result in the selection of different suitable HKGs in the six different tissues tested. Thus, different reference genes (*RPL32 *and *RPL13A*) should be used for the analysis of rhesus monkey tissue than those used for human tissue analysis

## Materials and methods

### RNA samples

Total RNA from rhesus macaque tissues (colon, liver, brain, lung, stomach, and kidney) and human total RNA (colon, liver, brain, lung, stomach, and kidney) were purchased from Clontech and Ambion, respectively. For the validation of our results for reference gene selection, corresponding unknown rhesus macaque tissues (colon, liver, brain, lung, stomach, and kidney) were provided by the National Primate Research Center (NPRC) of Korea. Total RNA was quantified using a NanoDrop^® ^ND-1000 UV-Vis Spectrophotometer.

### Rhesus macaque and human tissue cDNA synthesis

To eliminate DNA contamination from the total RNA samples, Turbo DNA-freeTM (Ambion) was used. A no-RT control was also amplified to confirm the absence of DNA contamination. M-MLV (Moloney-Murine Leukemia Virus) reverse transcriptase with an annealing temperature of 42°C was used for the reverse transcription reaction with RNase inhibiter (Promega).

### Candidate reference genes and primers for real-time quantitative RT-PCR

Rhesus monkey reference genes were identified by comparison with human HKGs using the NCBI (National Center for Biotechnology Information) database and the UCSC (University of California Santa Cruz) genome browser (Table 1). Their sequences were used to design primers design using Primer3 software [25]. BLAST searches were performed to confirm the total gene specificity of the primer sequences, and the results showed the absence of multi-locus matching at the primer site. All primers except for *RPL32 *spanned at least one intron to avoid inaccuracy due to genomic DNA contamination in RNA samples.

### Real-time quantitative RT-PCR

SYBR green qRT-PCR was performed on a Rotor Gene 3000 (Corbett Research). In each run, 1 ul of cDNA was used as template for amplification per reaction. The sample was added to 19 ul of reaction mixture containing 7 ul H_2_O, 10 ul QuantiTect^® ^SYBR^® ^Green PCR Master Mix (Qiagen) and 1 ul forward and reverse primers (Table 2). Real-time qRT-PCR amplification of the HKGs was carried out for 50 cycles of 94°C for 10 sec, 58°C for 15 sec, and 72°C for 15 sec. The temperature range for the analysis of melting curves was 55°C to 99°C over 30 sec. Three independent experiments were performed.

### Characterization of analysis programs

The *geNorm *[22] program provides a measure of gene expression stability (M), which is the mean pair-wise variation between an individual gene and all other tested control genes. This method differs from model-based approaches by comparing genes based on the similarity of their expression profiles. Ct values were converted to scale expression quantities using the delta-Ct method and entered into *geNorm*, which then ranks the genes based on M values where the gene with the most stable expression has the lowest value. *NormFinder *[23] focuses on finding the two genes with the least intra- and inter-group expression variation. A *BestKeeper *[24] index was created using the geometric mean of each candidate gene's Ct values. This index was then compared to each individual candidate housekeeping gene by pair-wise correlation analyses, with each combination assigned a value for the Pearson correlation coefficient (r) and the probability (p).

## Supplementary Material

Additional file 1 **Standard curves for calculation of PCR efficiency and quantification according to reference genes in the rhesus monkey**. Amplification of 10-fold serial dilutions of the plasmid standard ranging from 10^0 ^to 10^5 ^copies per reaction was carried out in duplicate. Click here for file

Additional file 2 **Standard curves for calculation of PCR efficiency and quantification according to reference genes in humans**. Amplification of 10-fold serial dilutions of the plasmid standard ranging from 10^0 ^to 10^5 ^copies per reaction was carried out in duplicate. Click here for file

Additional file 3 Efficiencies calculated from the slopes of dilution curves. Click here for file

Additional file 4 **Melting curve analysis**. Melting curve analysis of 8 different primer sets for reference genes in the rhesus monkey. Click here for file

Additional file 5 **Melting curve analysis**. Melting curve analysis of 8 different primer sets for reference genes in humans. Click here for file

Additional file 6 Selection of the most suitable reference genes for normalization using *geNorm *analysis using the unknown rhesus monkey samples for validation. Click here for file

Additional file 7 Candidate reference genes for normalization and the best combination of two genes listed according to their expression stability calculated by the NormFinder program using the unknown rhesus monkey samples for validation. Click here for file

Additional file 8 Results from Bestkeeper descriptive statistical analysis using the unknown rhesus monkey samples for validation. Click here for file
