# Supplementary figures and images for: Selection of internal reference genes for SYBR green qRT-PCR studies of rhesus monkey (Macaca mulatta) tissues
Source: BMC Mol Biol. 2008 Sep 10;9:78. doi: 10.1186/1471-2199-9-78 (PMC2561044; doi:10.1186/1471-2199-9-78)

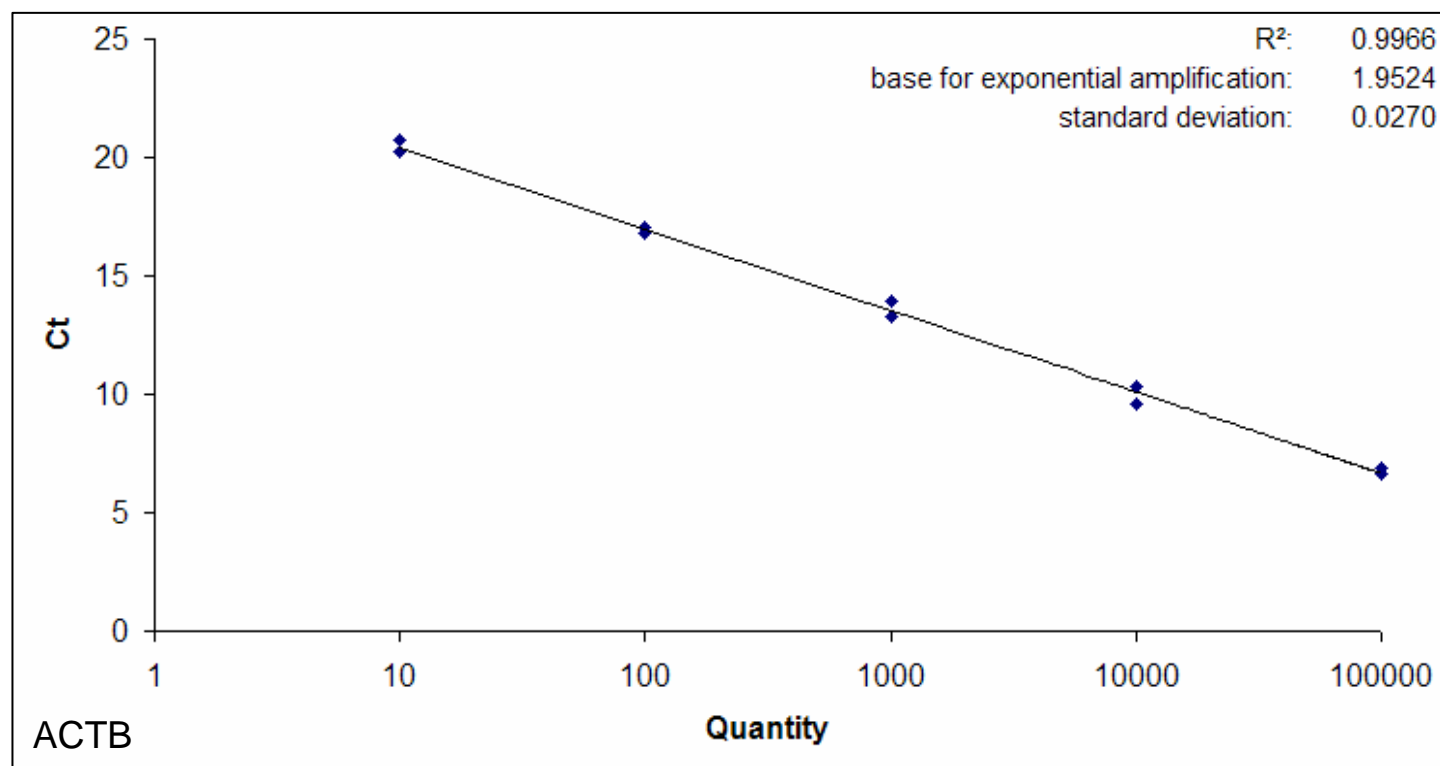

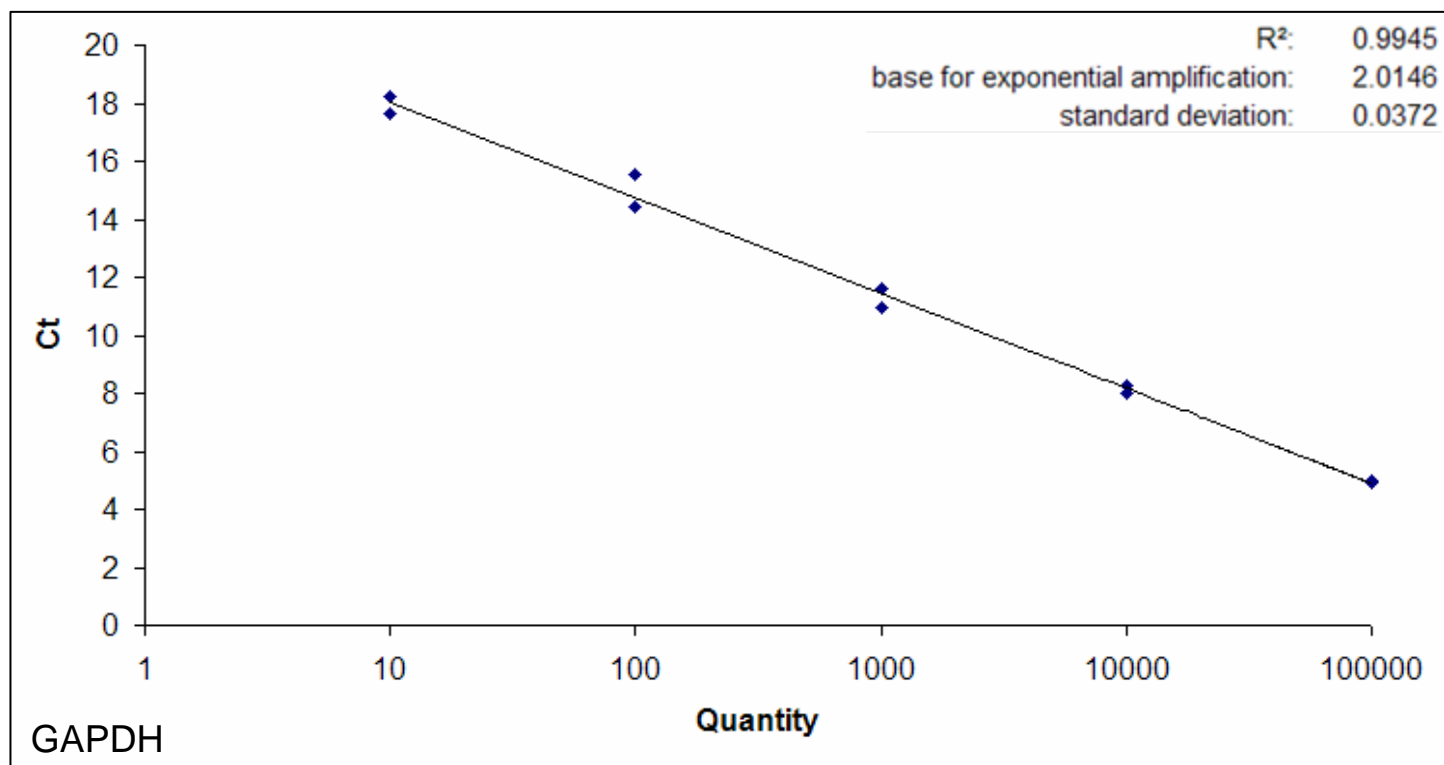

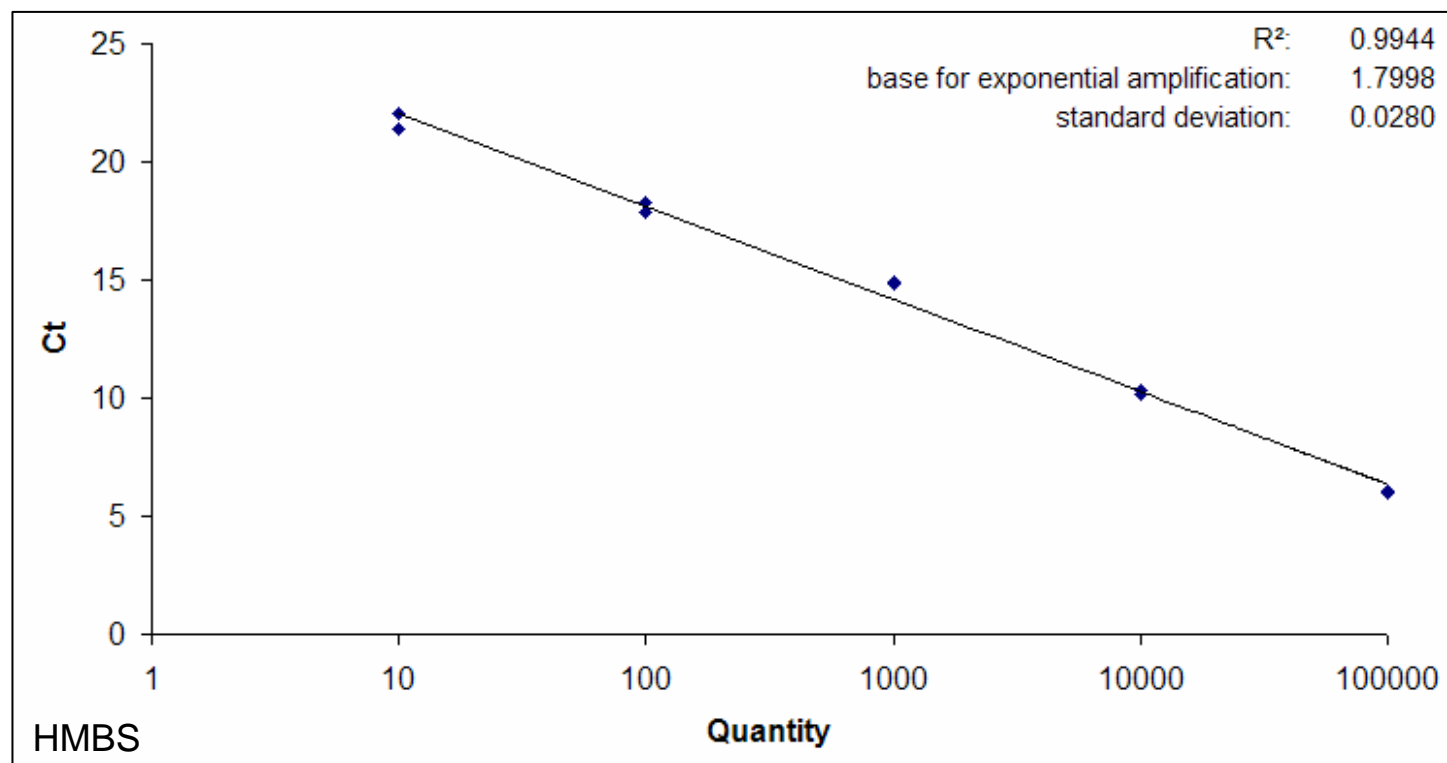

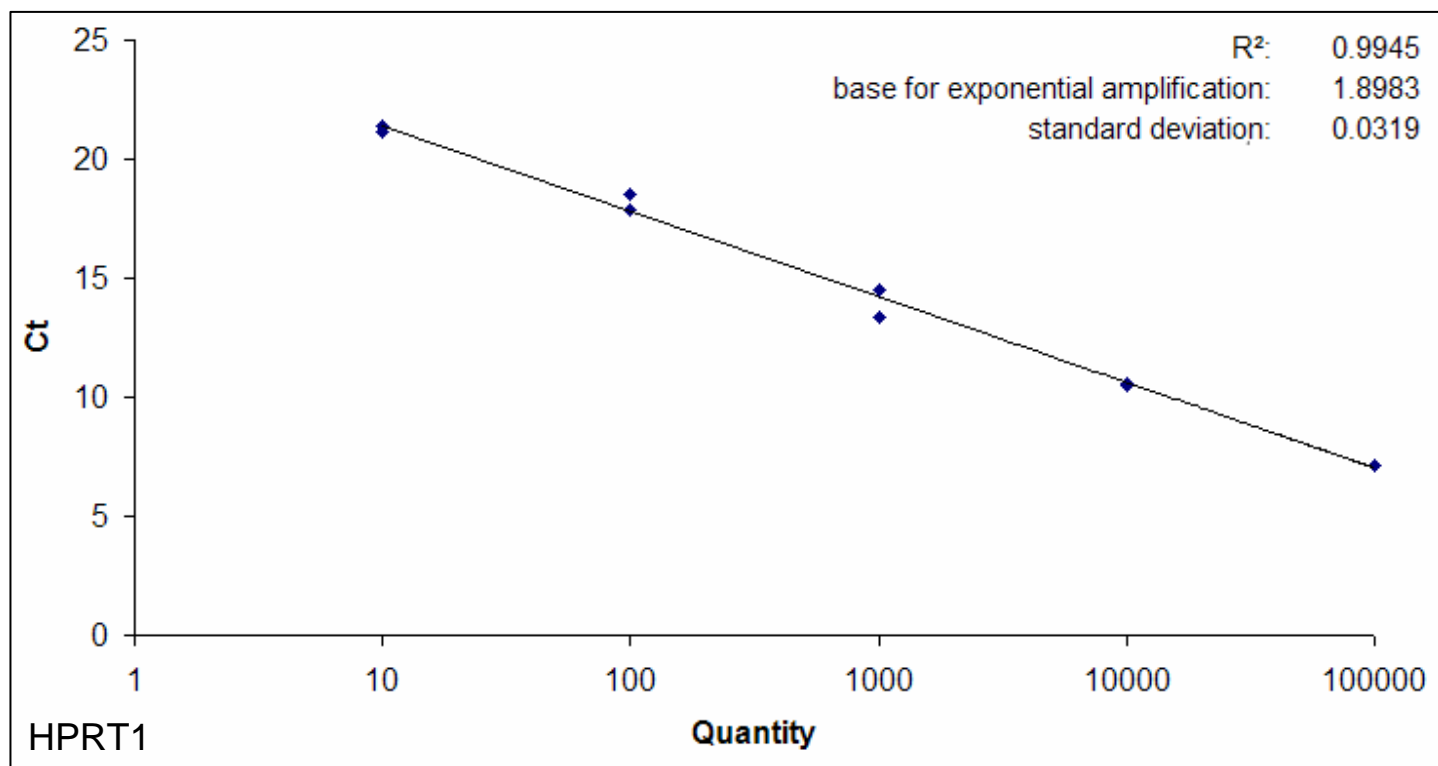

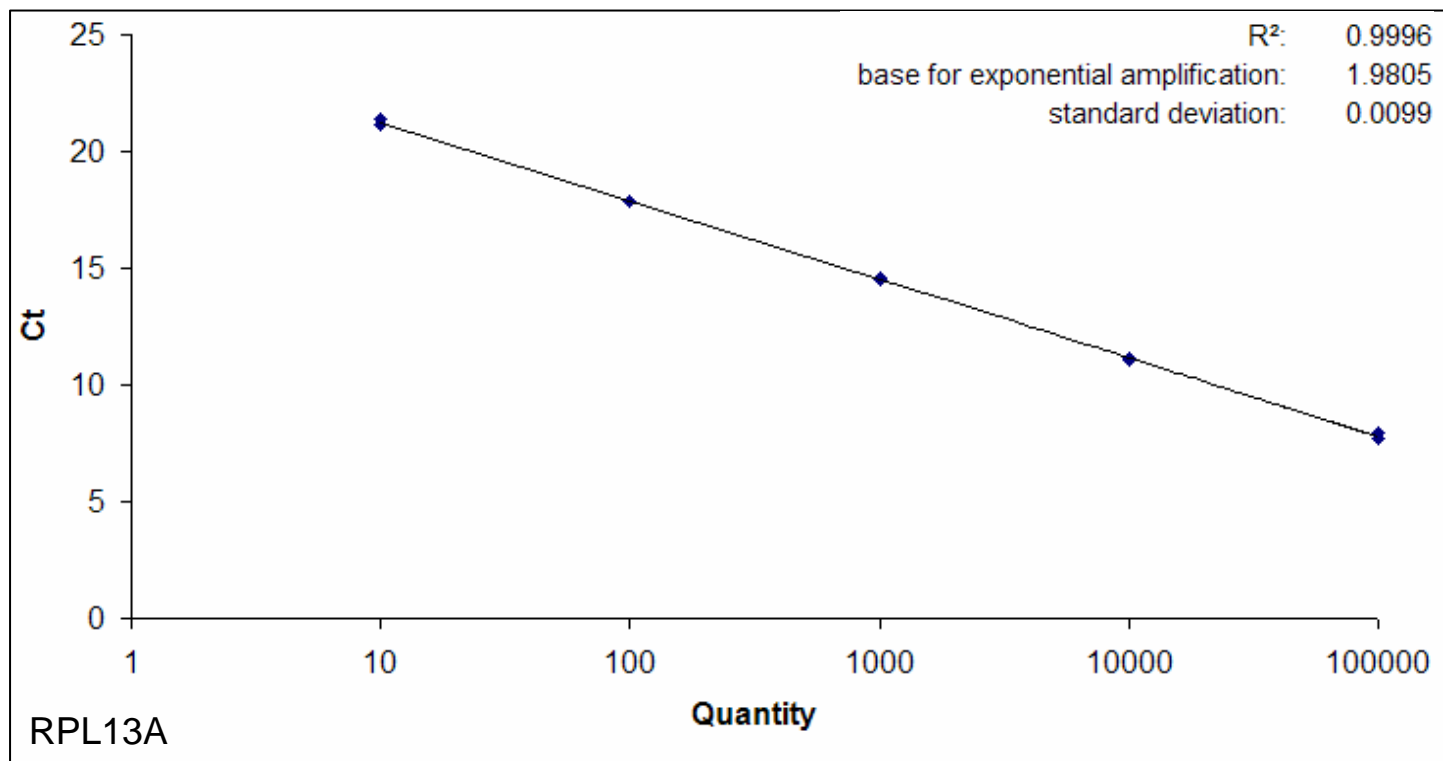

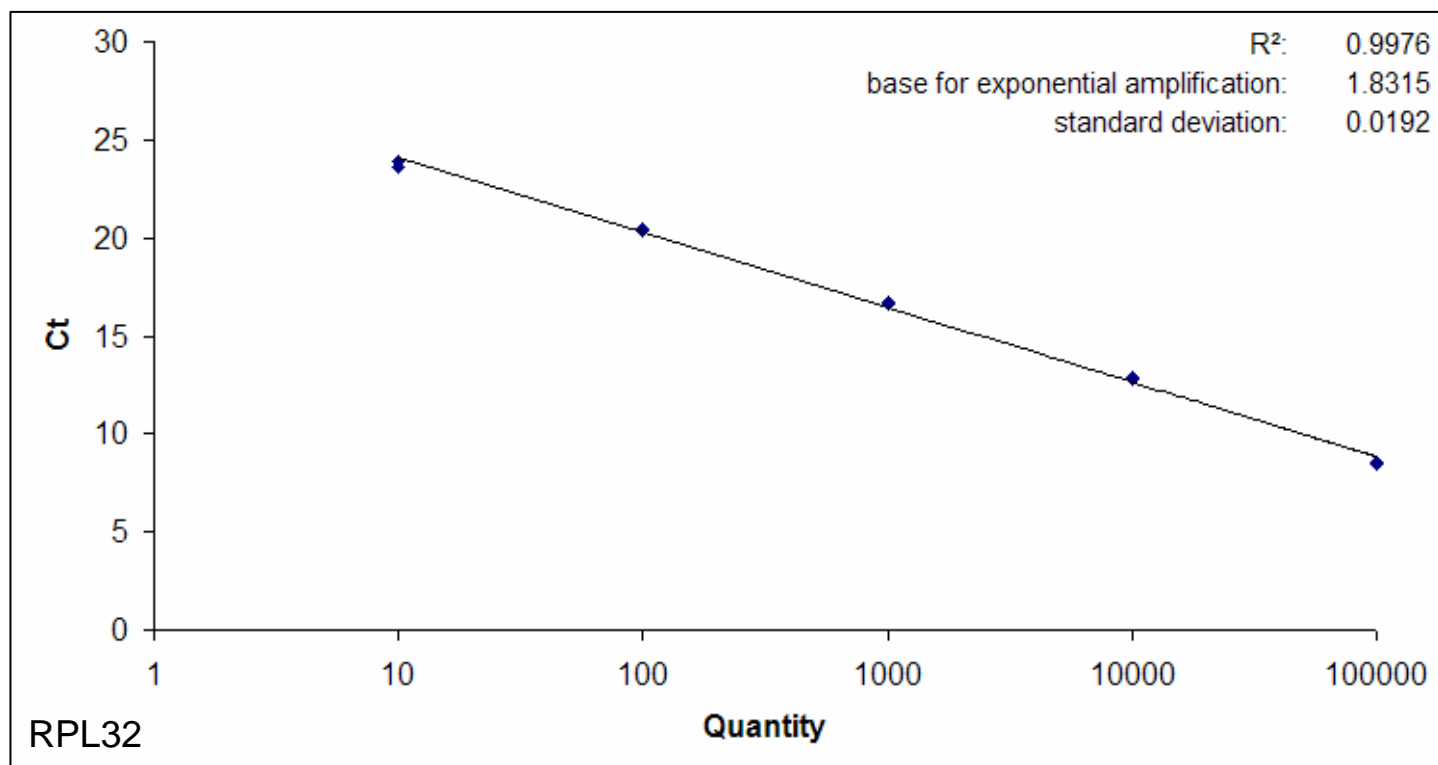

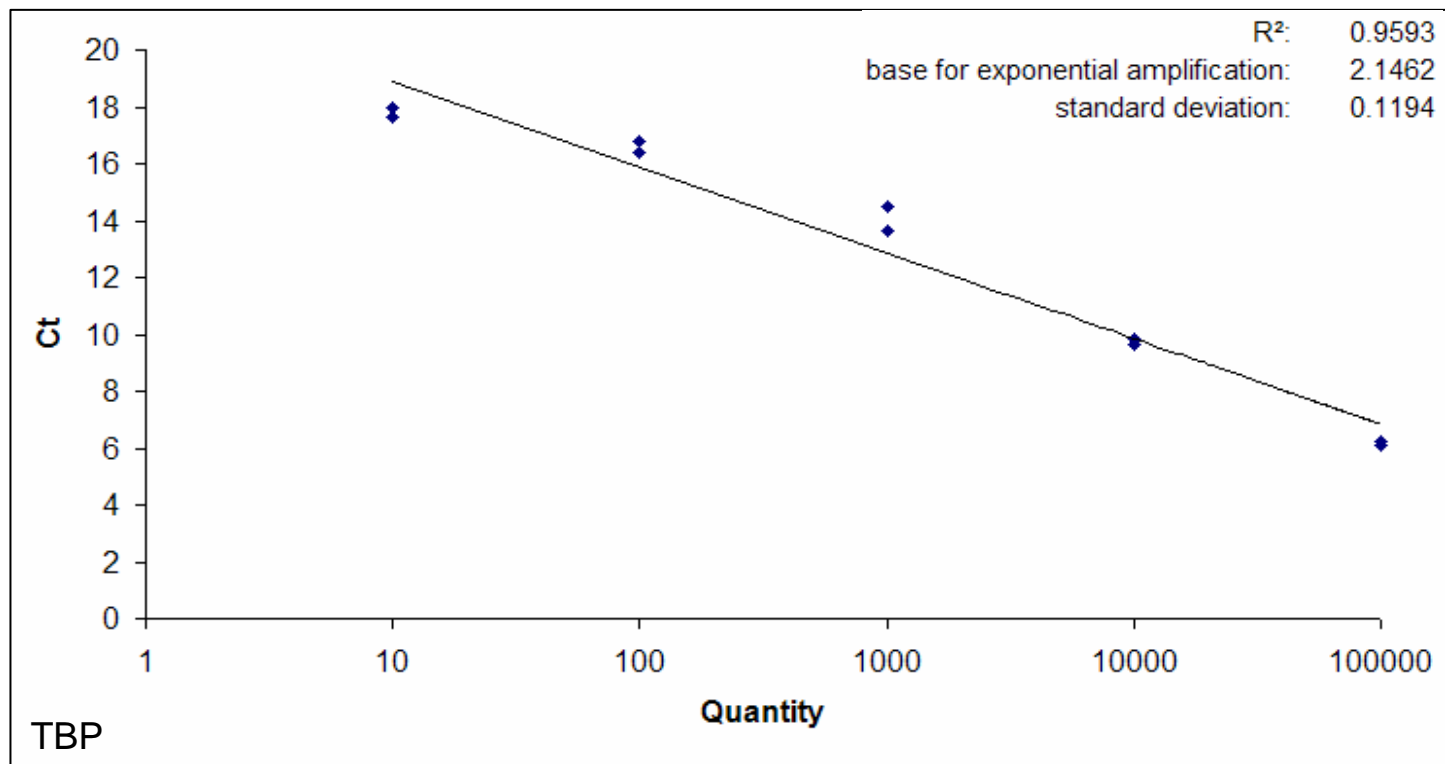

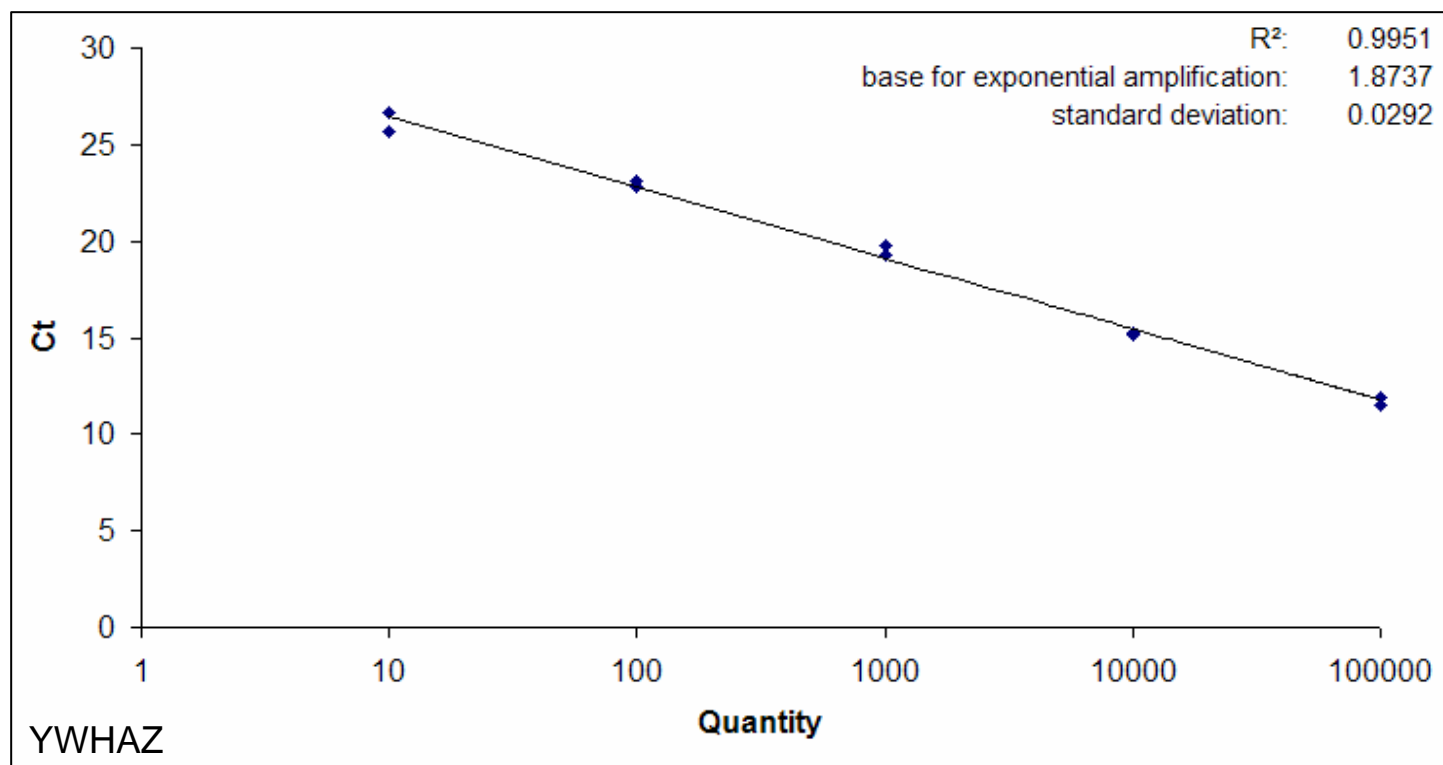

Supplement: Additional file 1 — Standard curves for calculation of PCR efficiency and quantification according to reference genes in the rhesus monkey. Amplification of 10-fold serial dilutions of the plasmid standard ranging from 100 to 105 copies per reaction was carried out in duplicate. [file 1471-2199-9-78-S1.pdf]

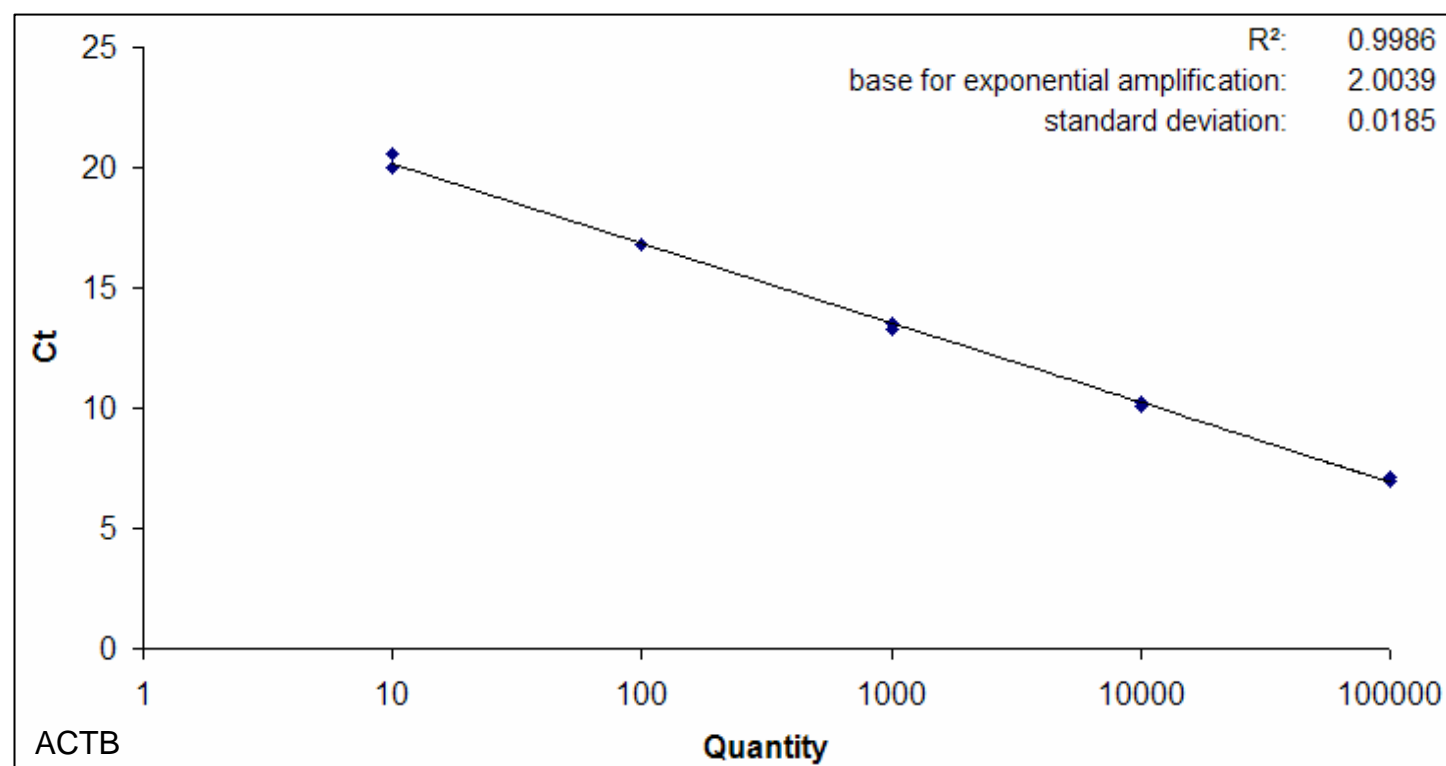

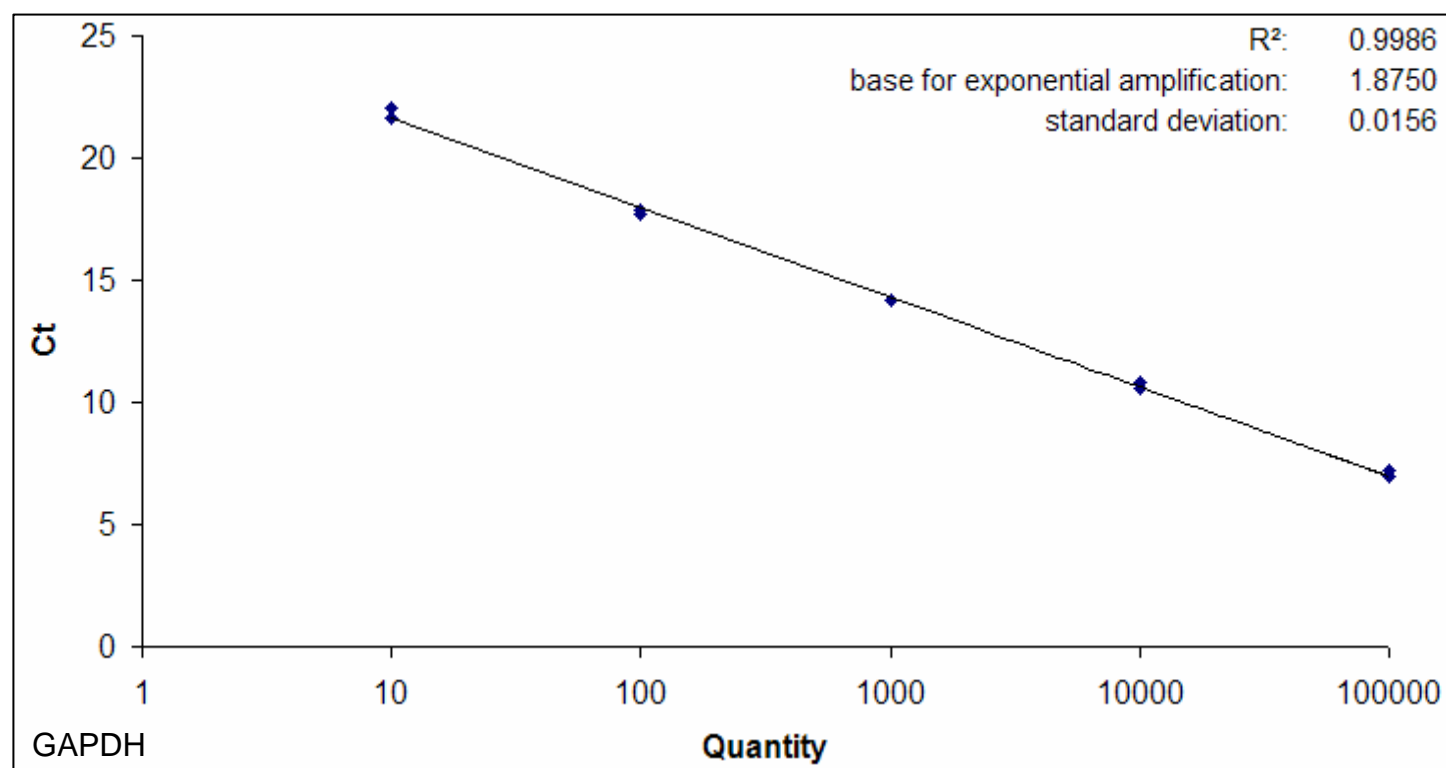

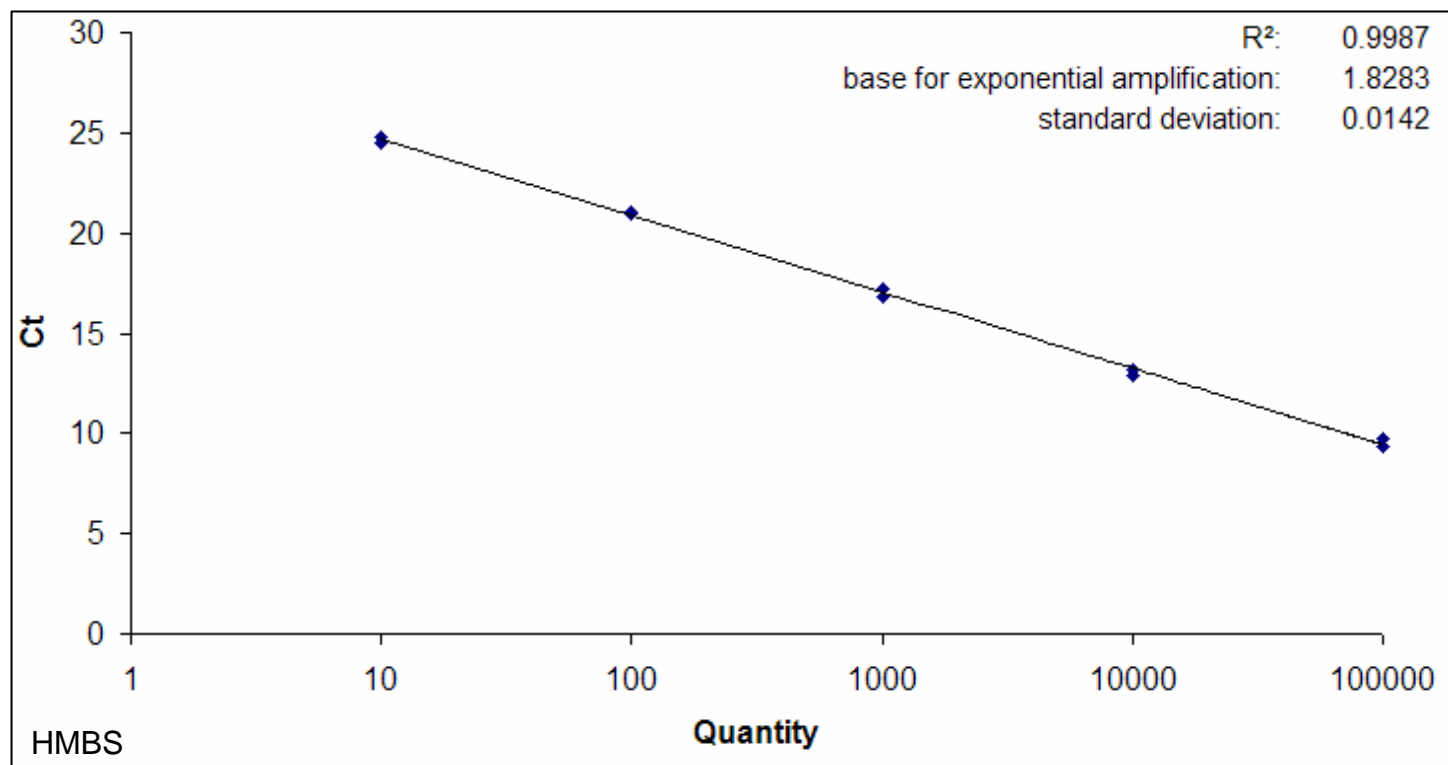

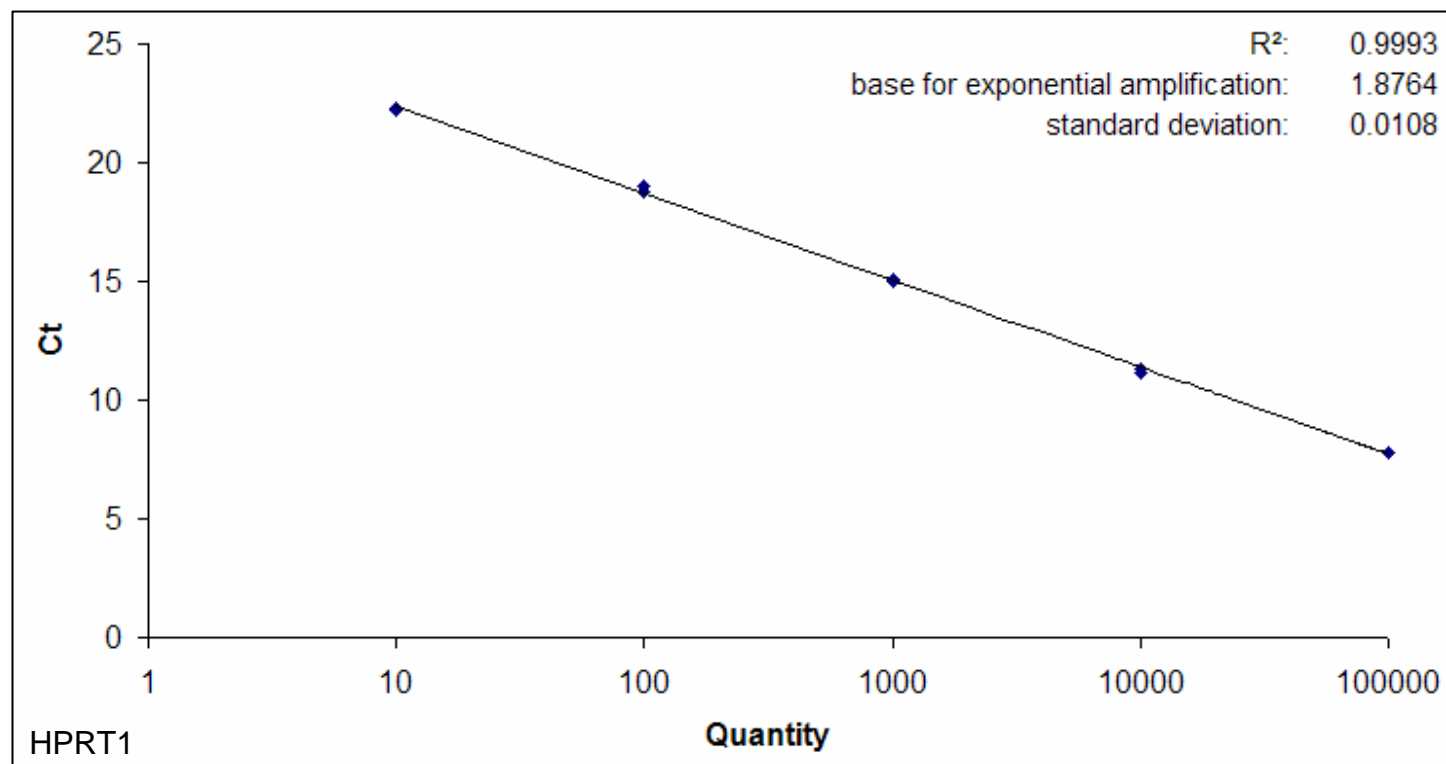

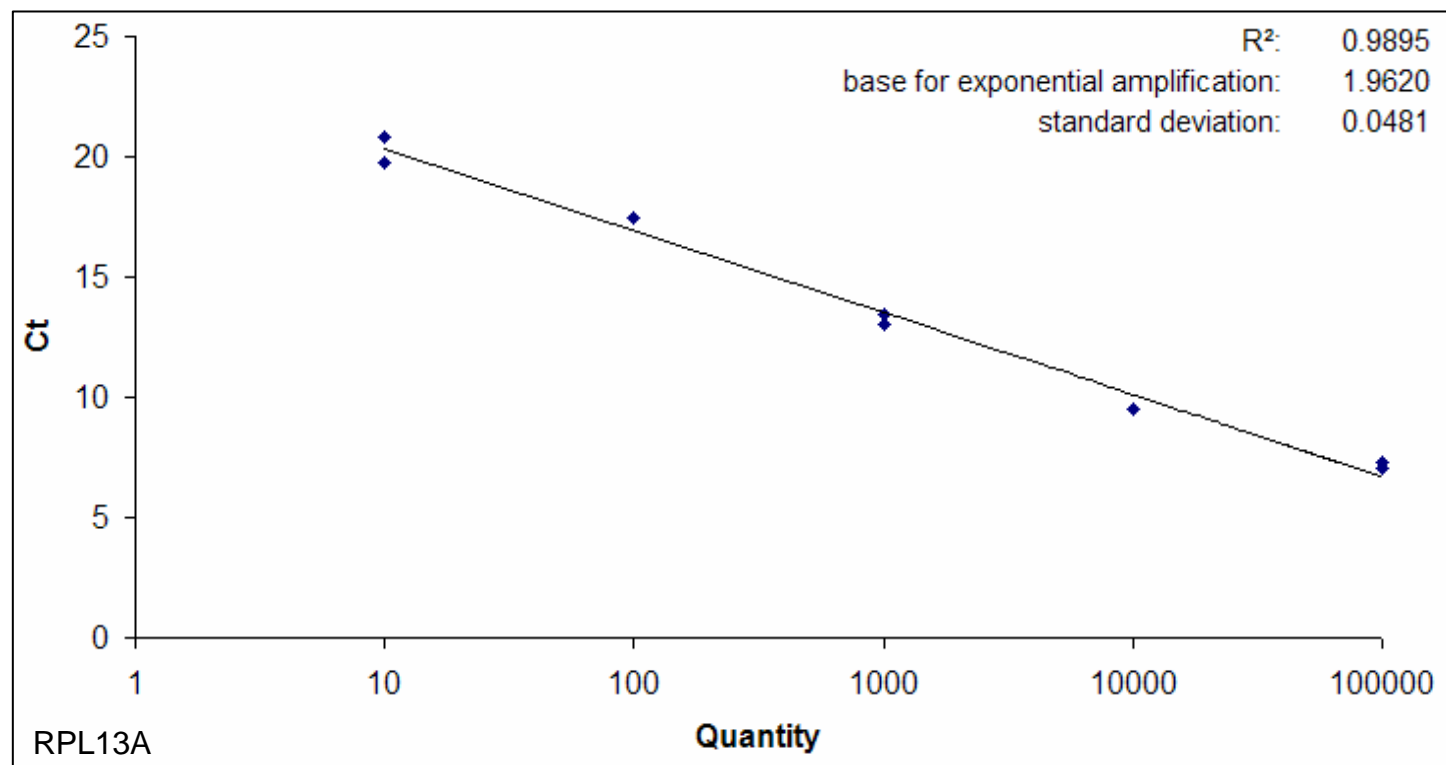

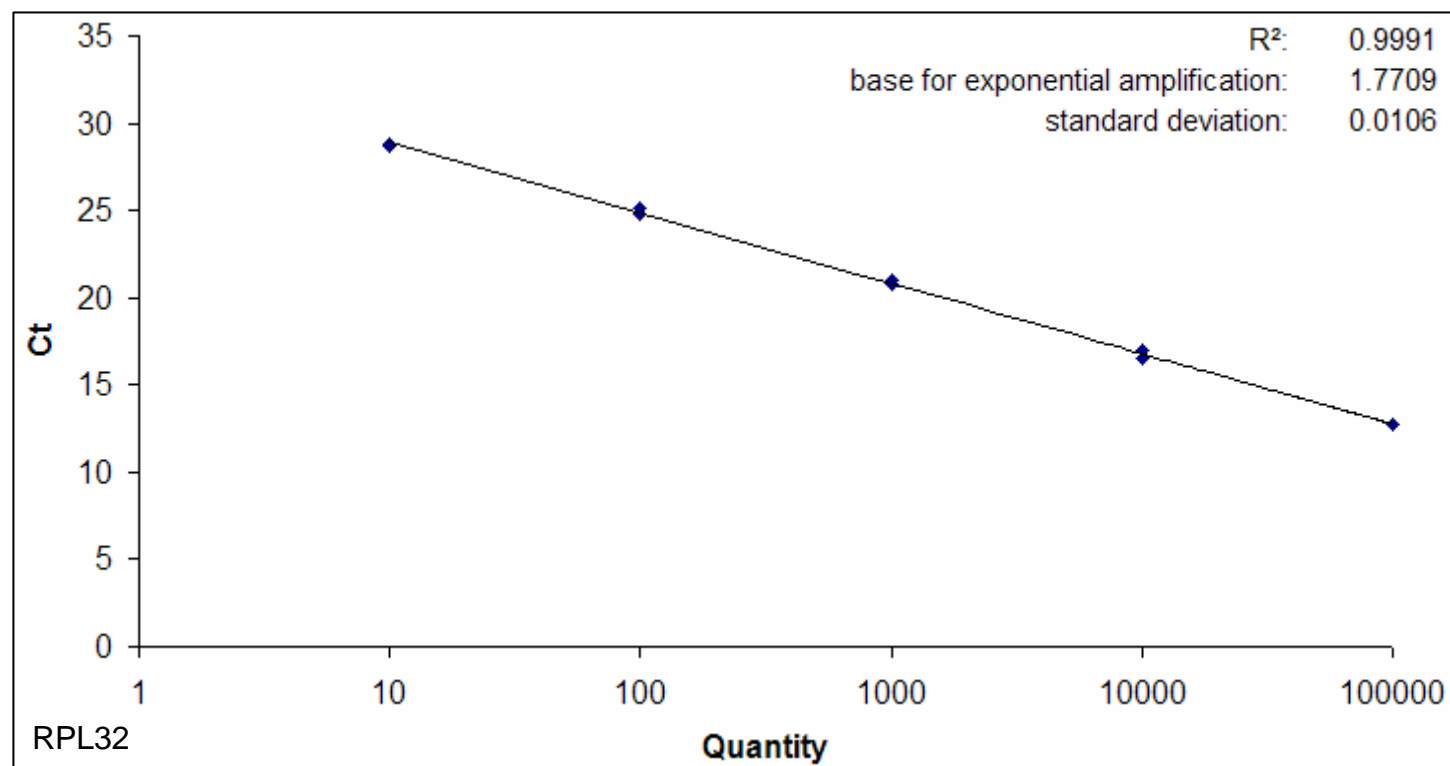

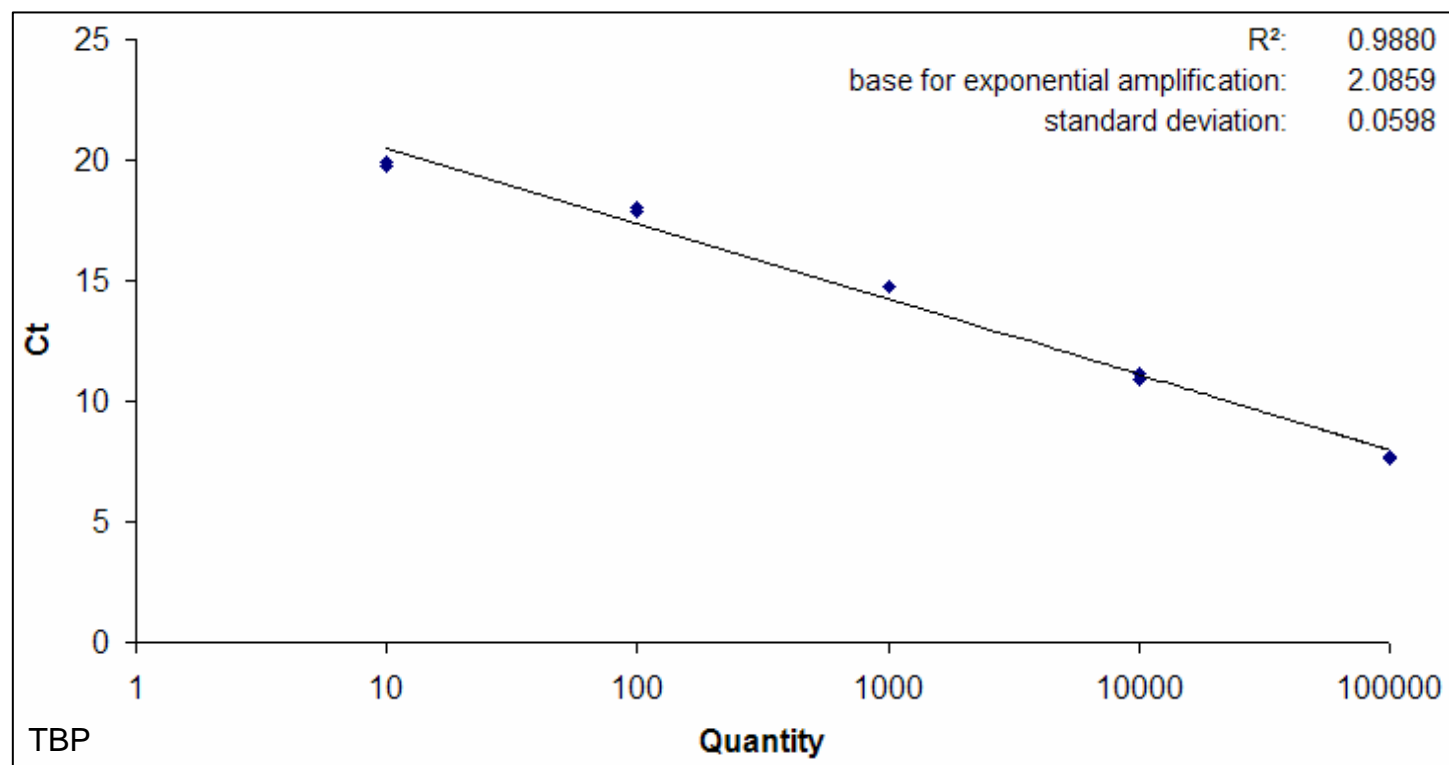

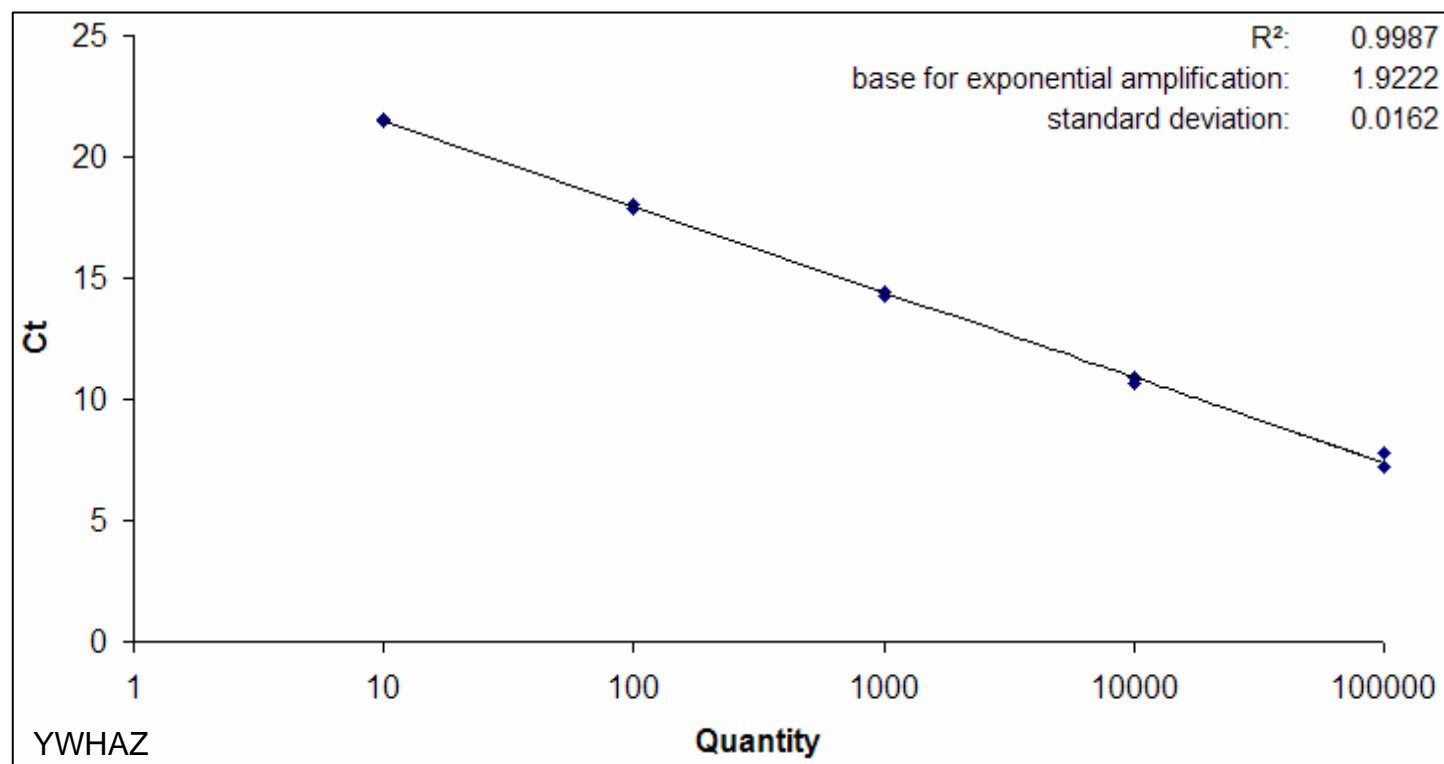

Supplement: Additional file 2 — Standard curves for calculation of PCR efficiency and quantification according to reference genes in humans. Amplification of 10-fold serial dilutions of the plasmid standard ranging from 100 to 105 copies per reaction was carried out in duplicate. [file 1471-2199-9-78-S2.pdf]

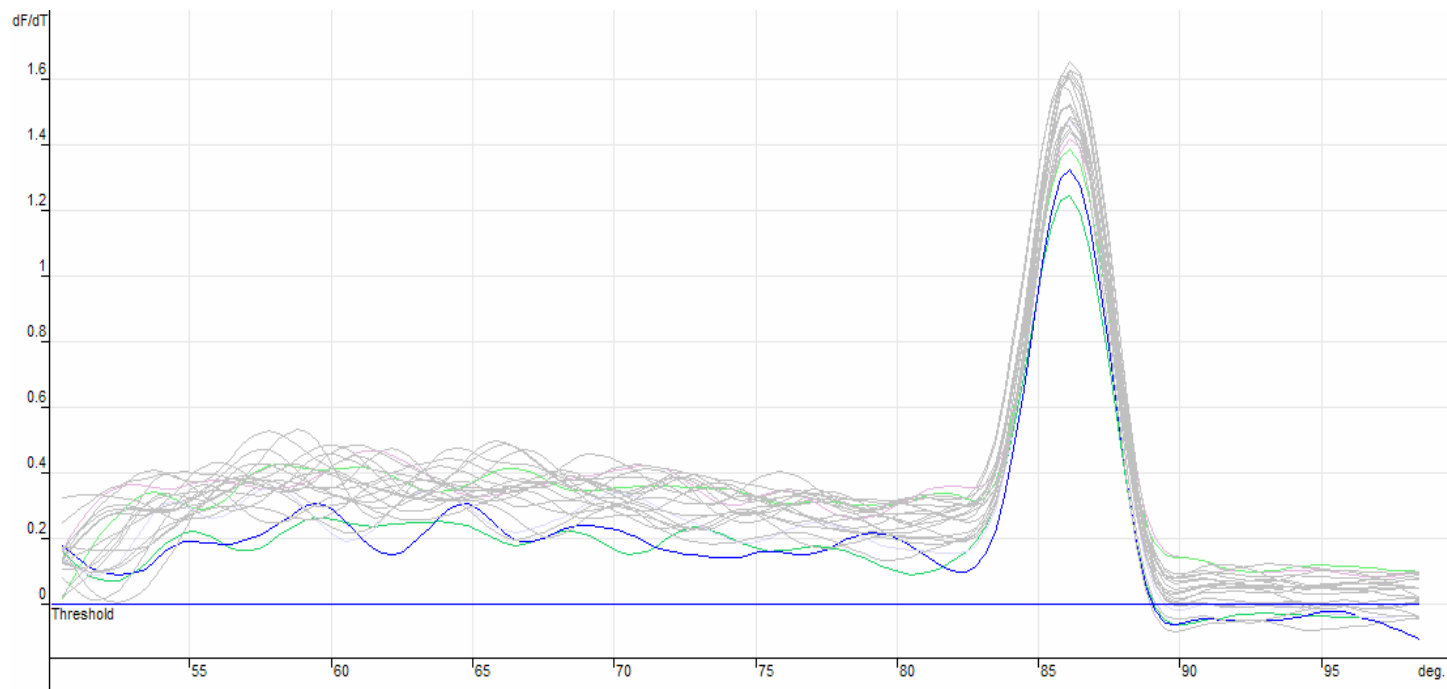

ACTB

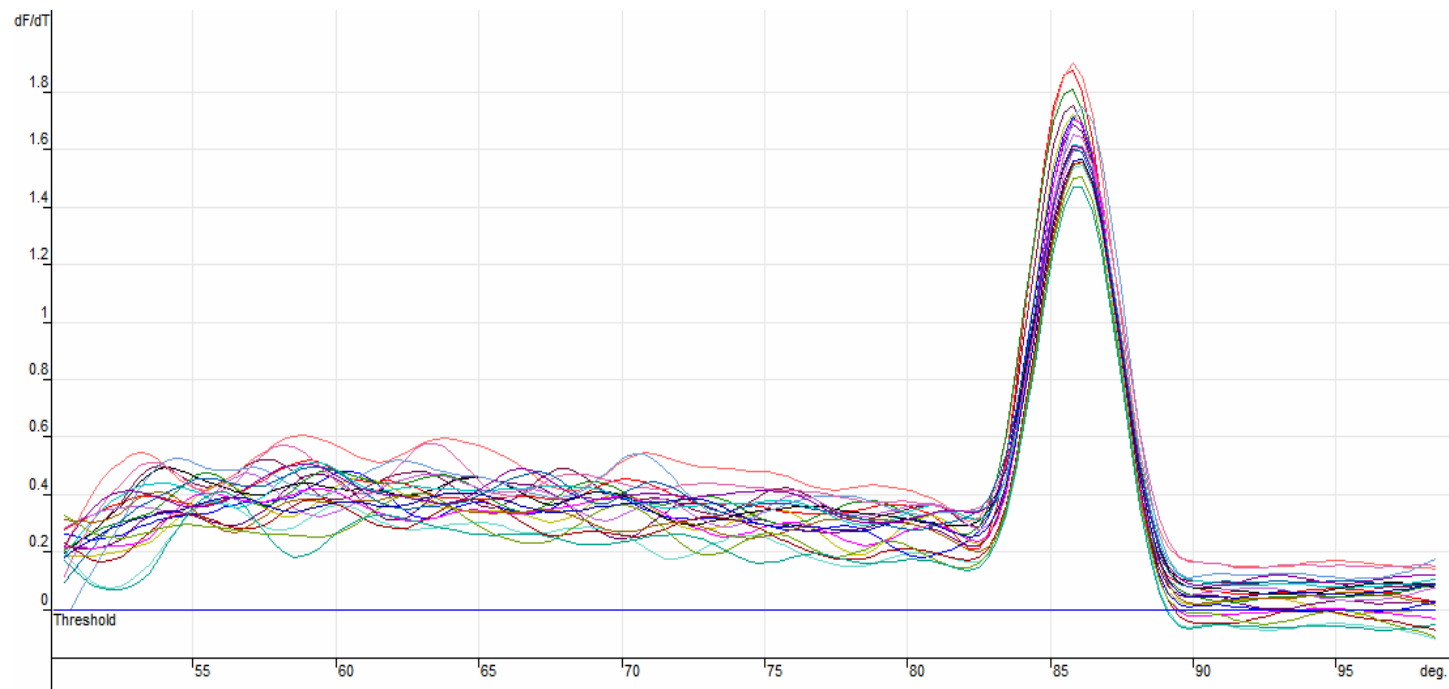

GAPDH

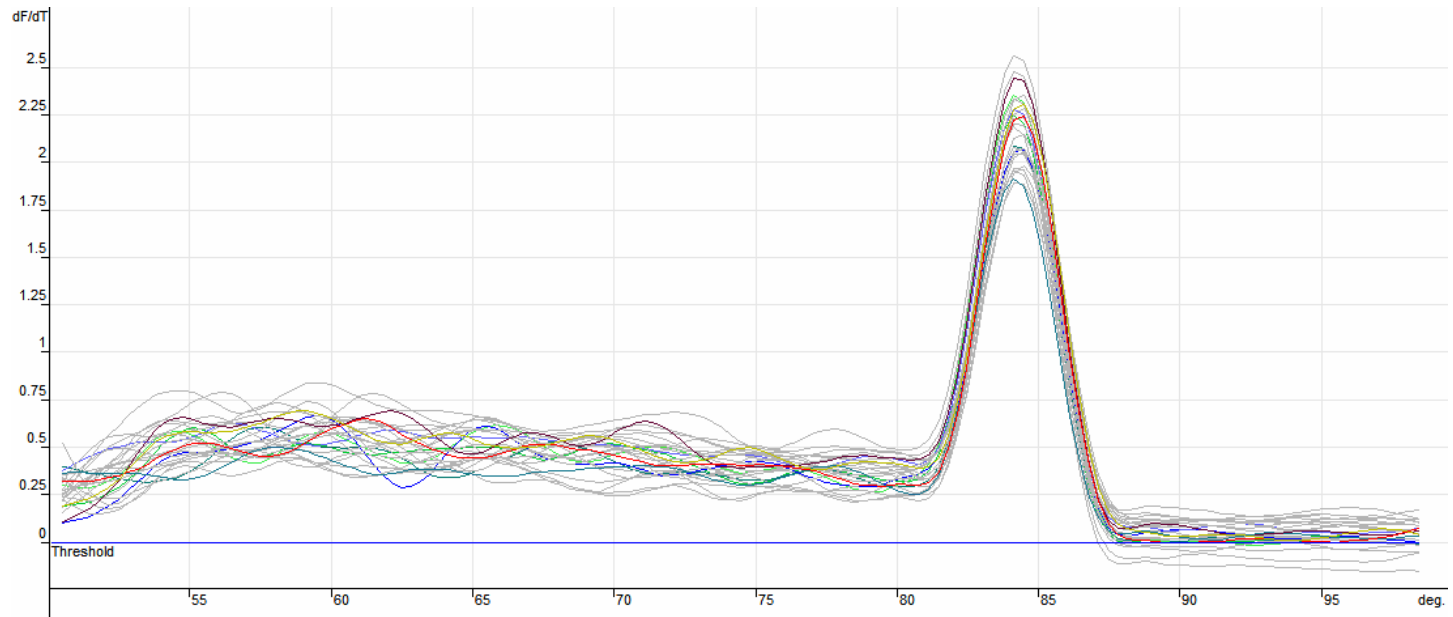

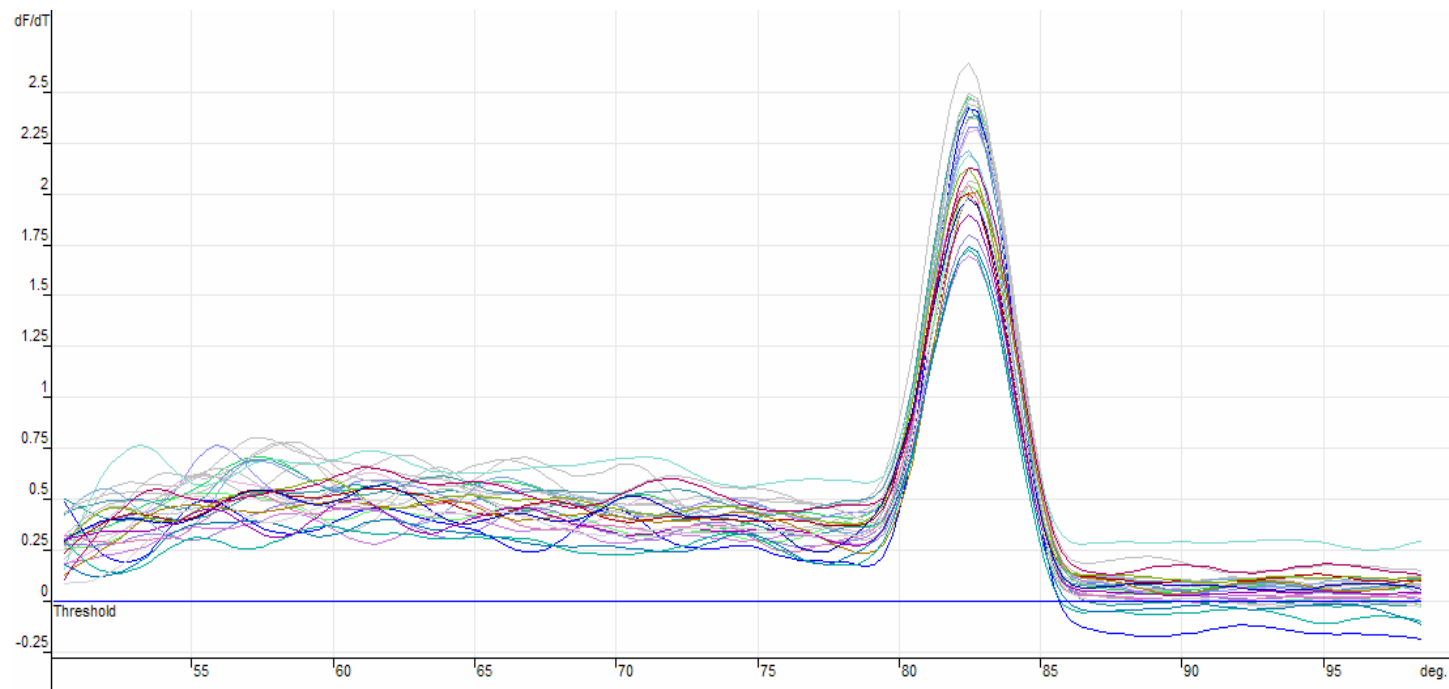

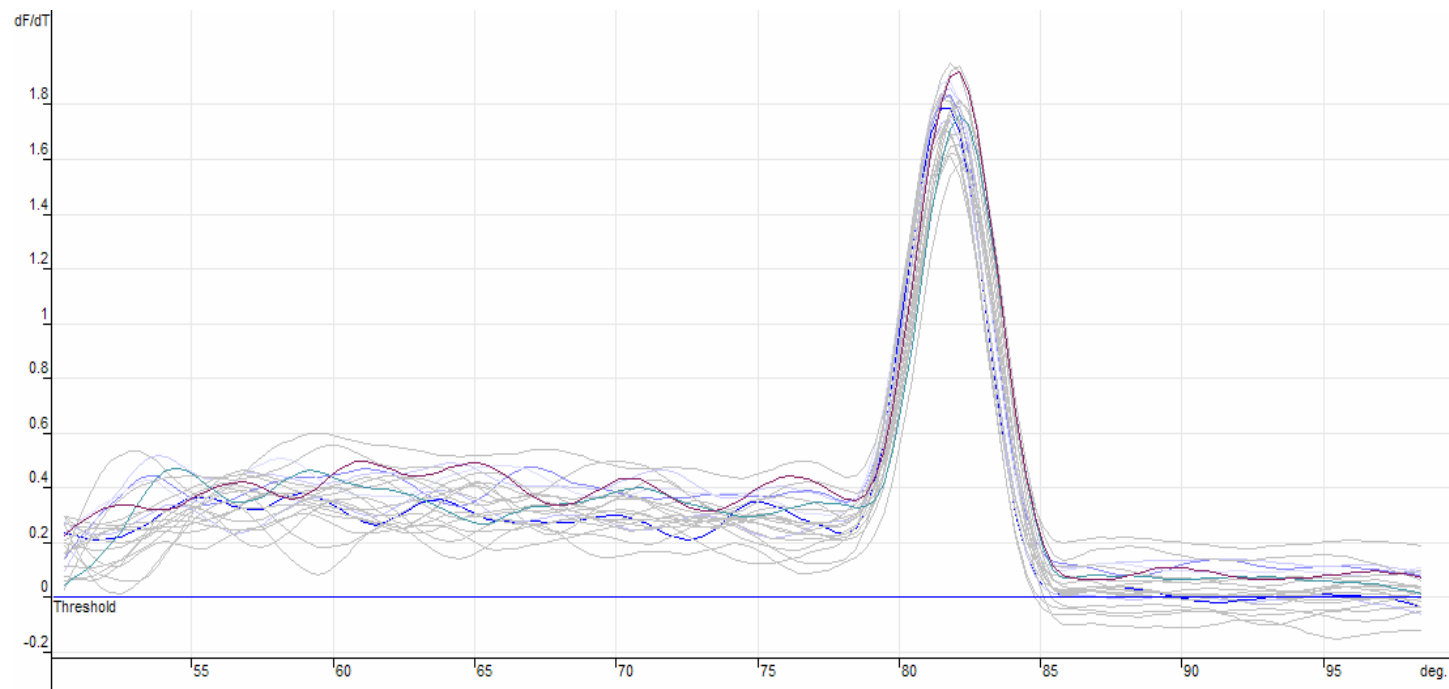

RPL13A

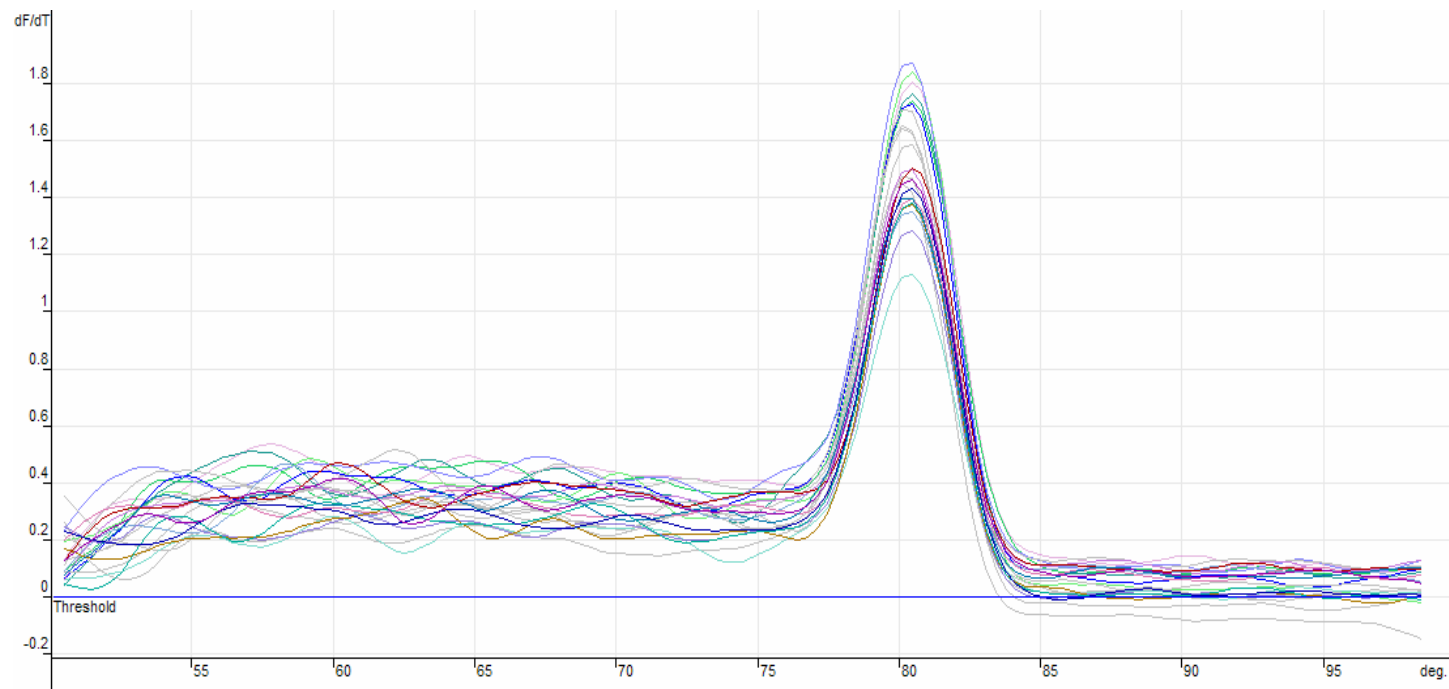

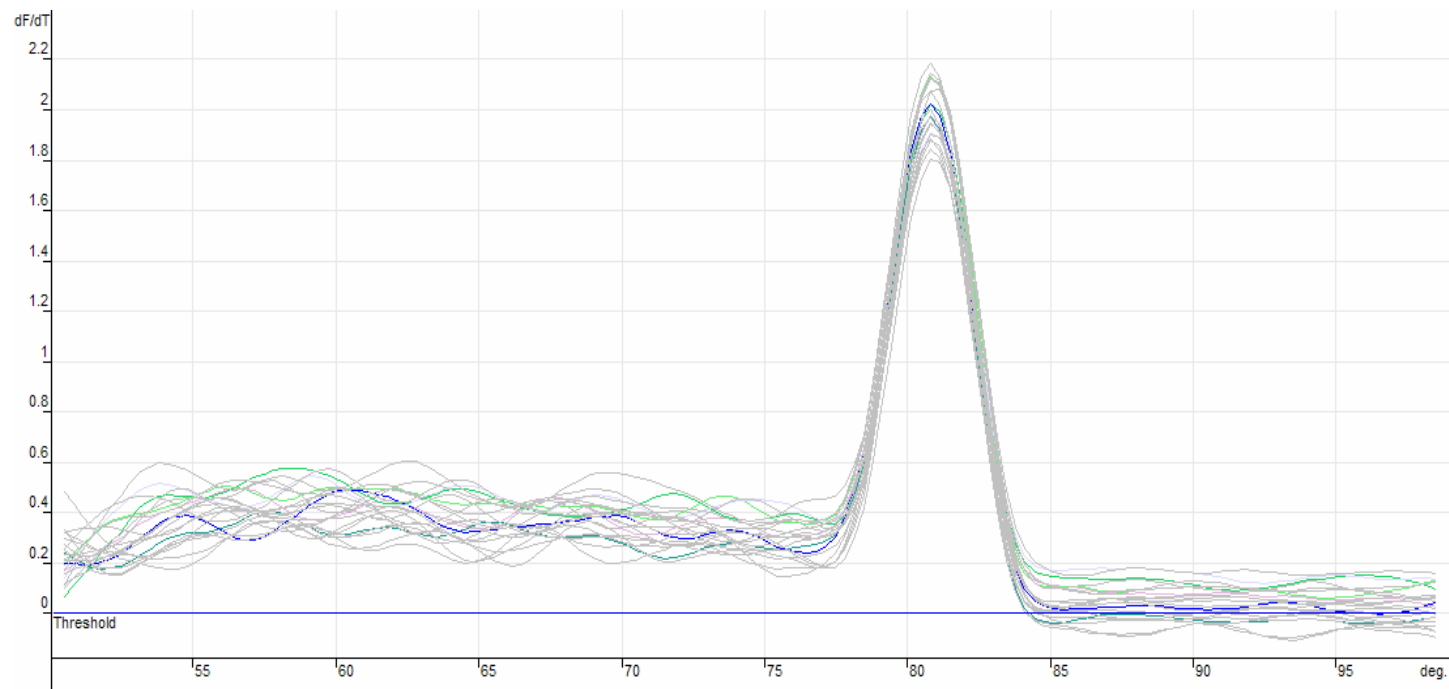

TBP

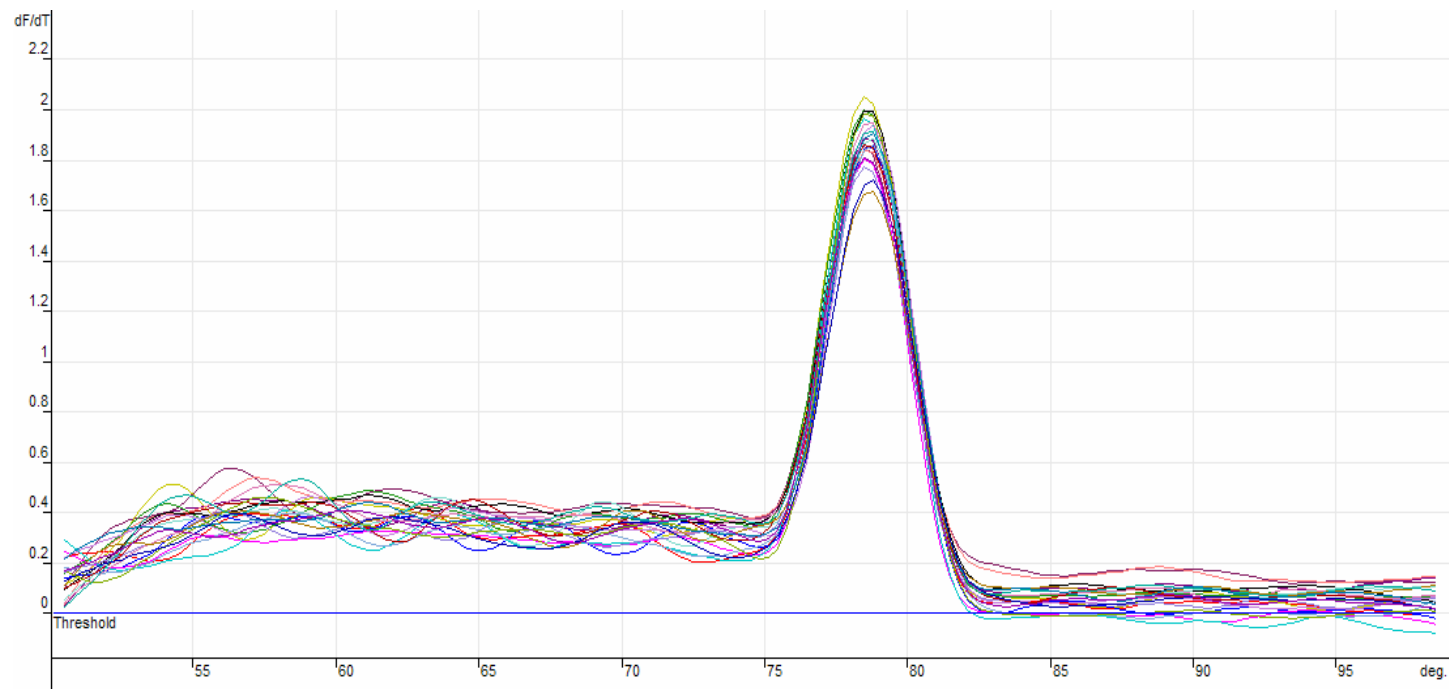

YWHAZ

Supplement: Additional file 4 — Melting curve analysis. Melting curve analysis of 8 different primer sets for reference genes in the rhesus monkey. [file 1471-2199-9-78-S4.pdf]

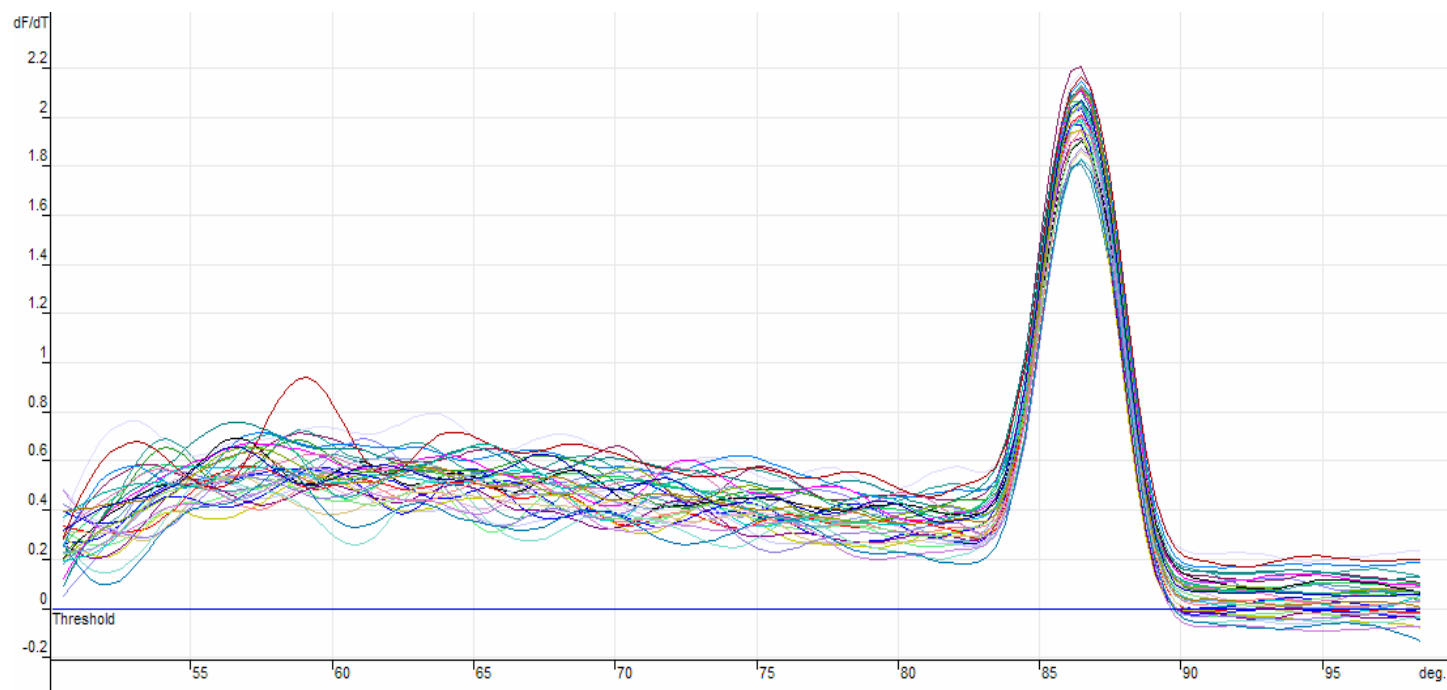

ACTB

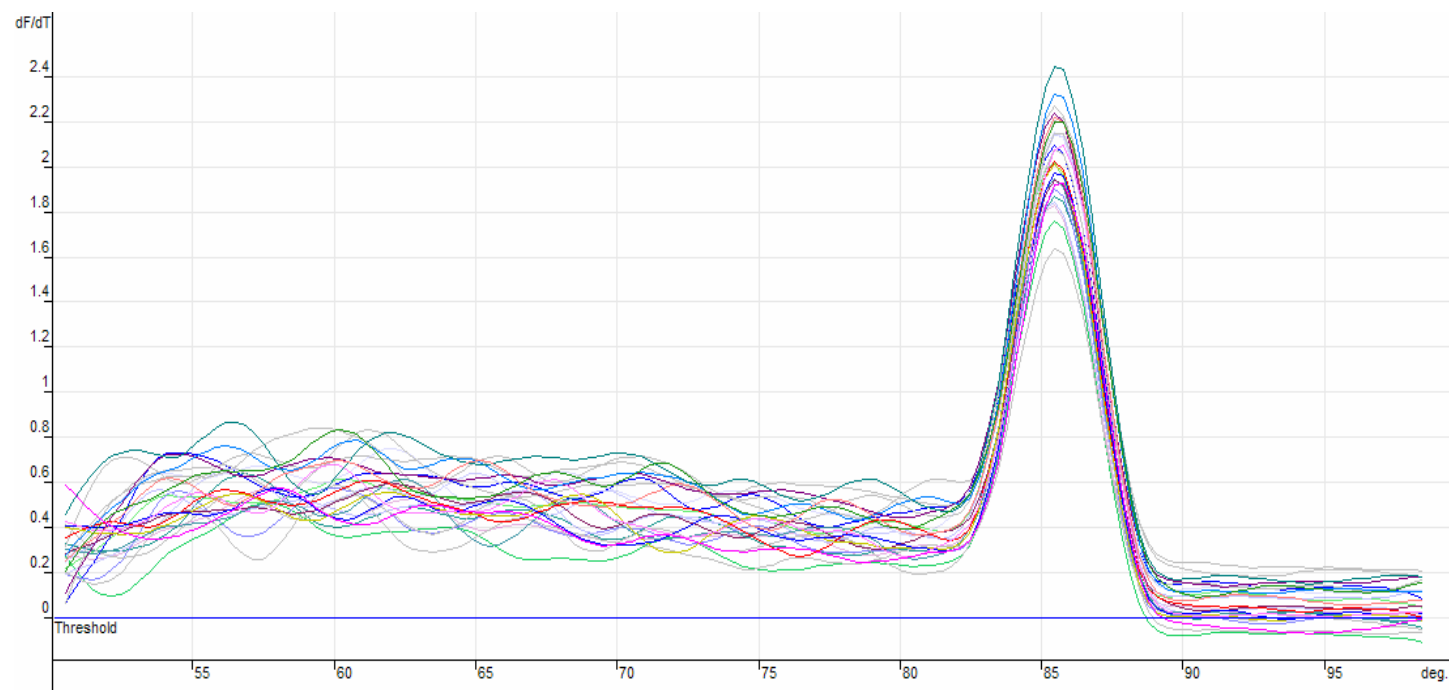

GAPDH

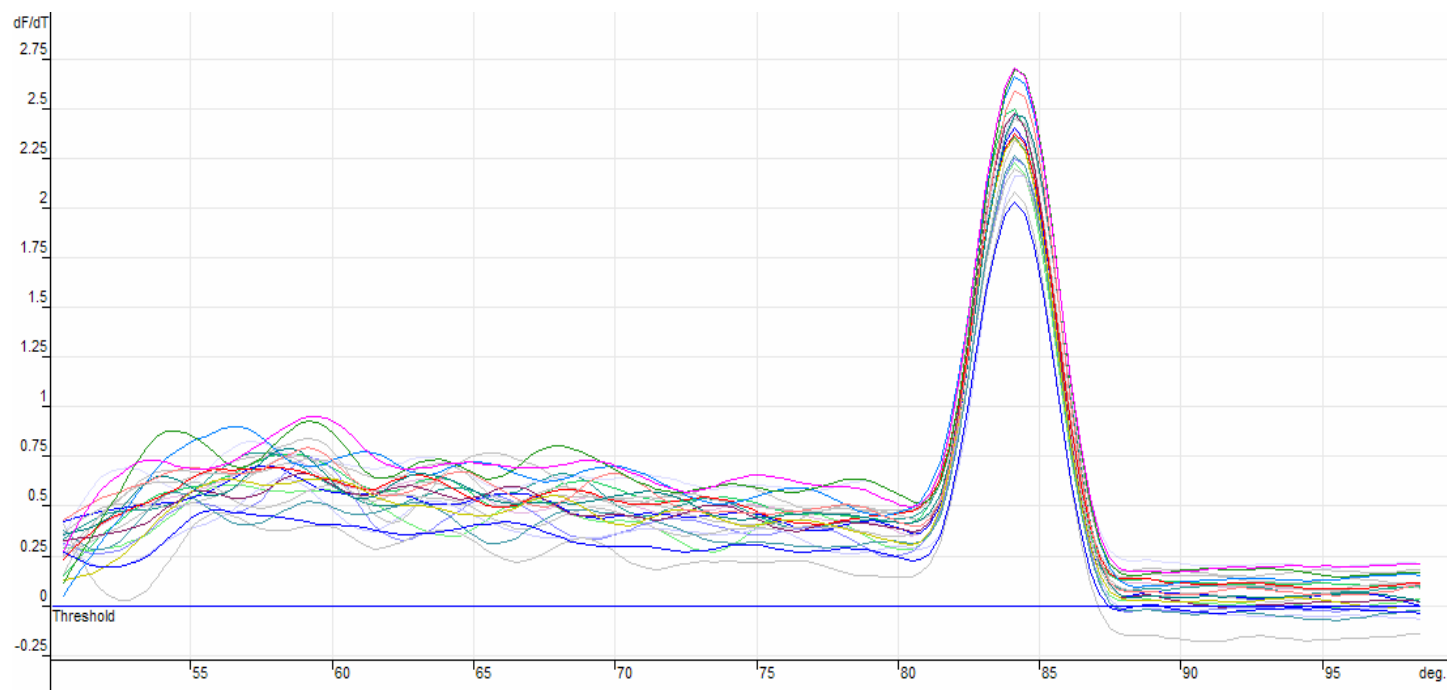

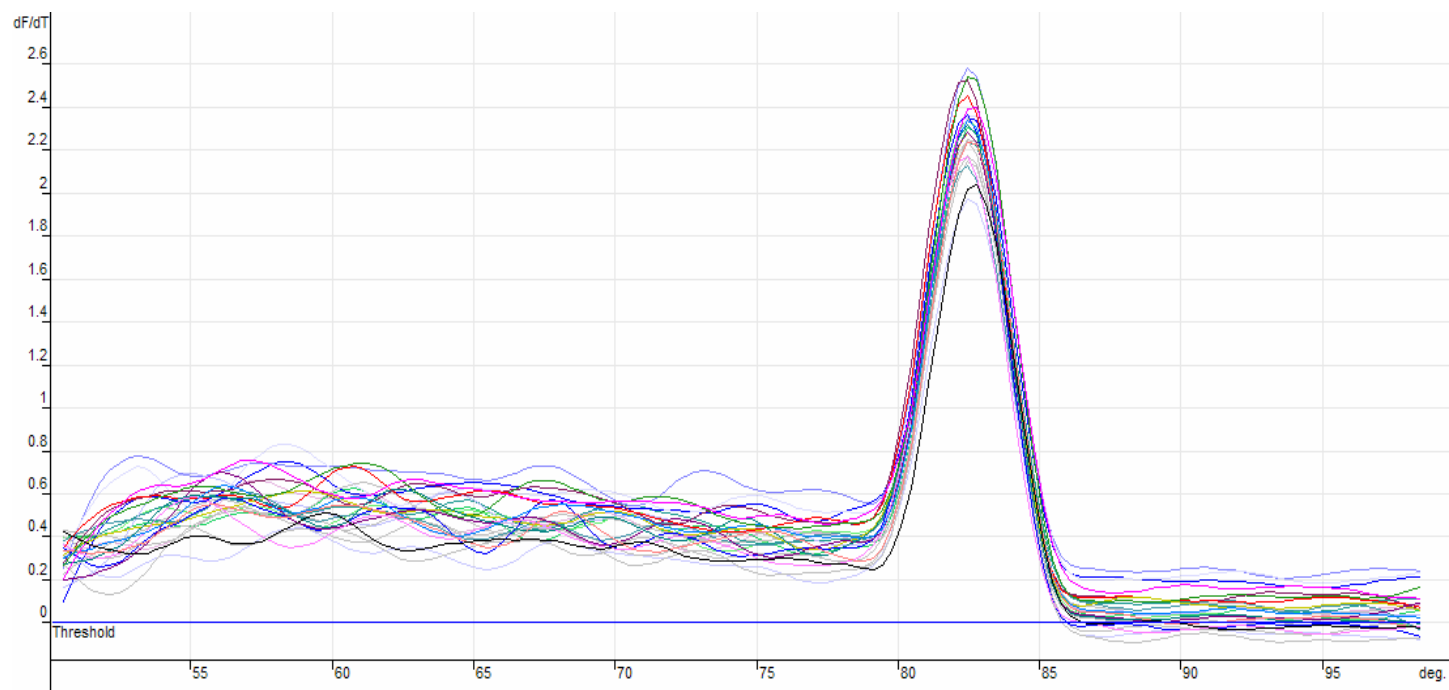

HPRTI

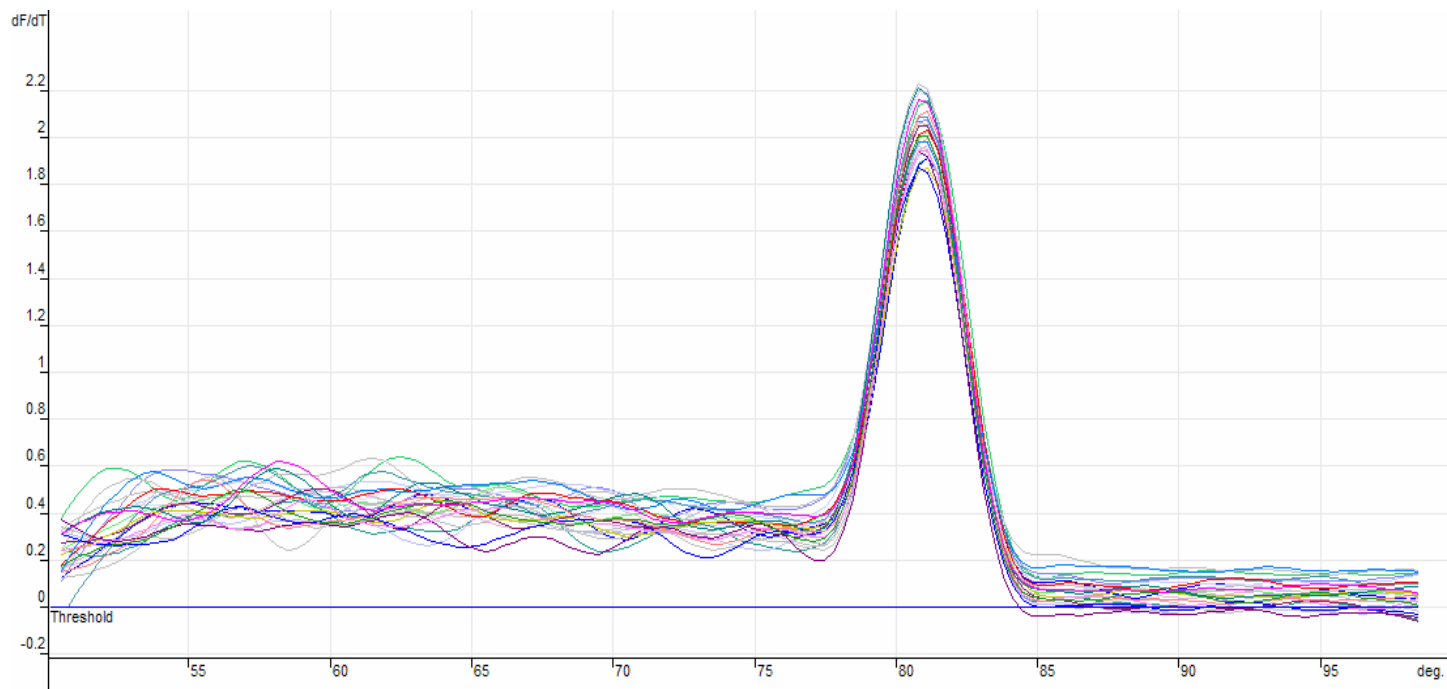

RPL13A

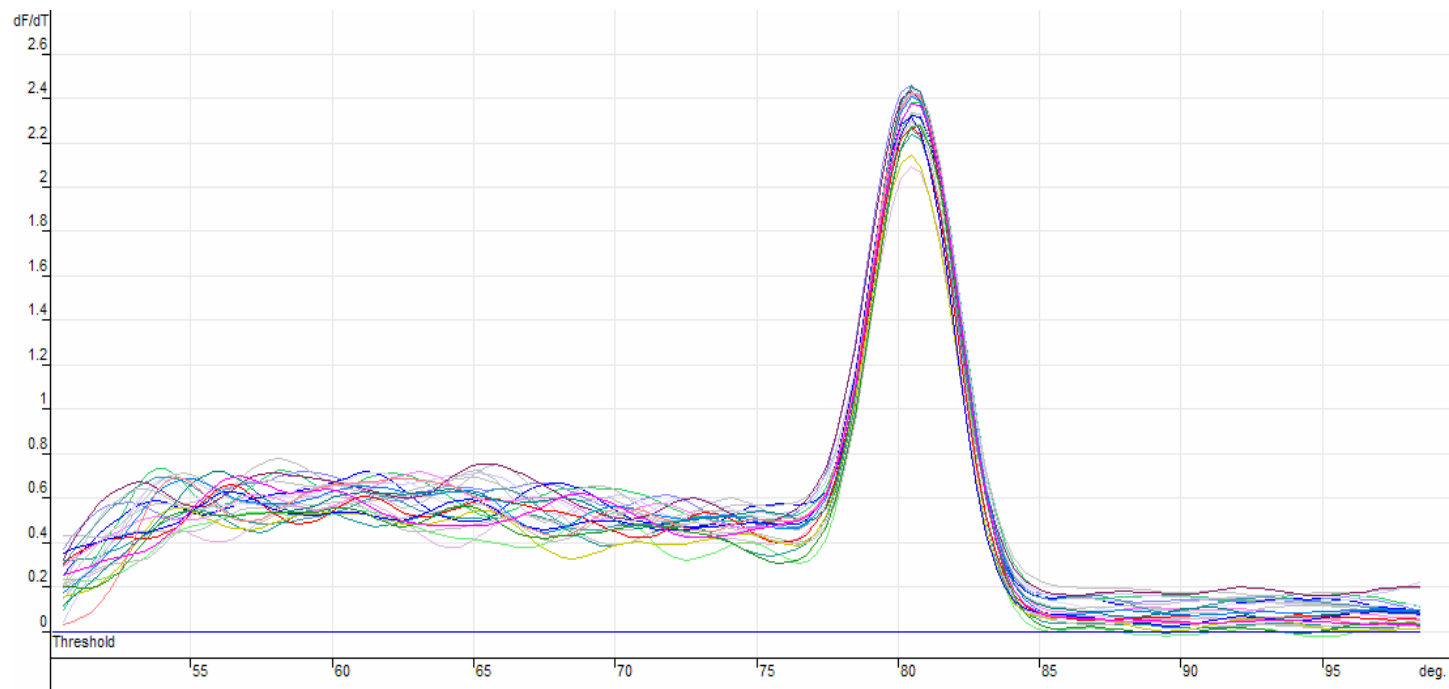

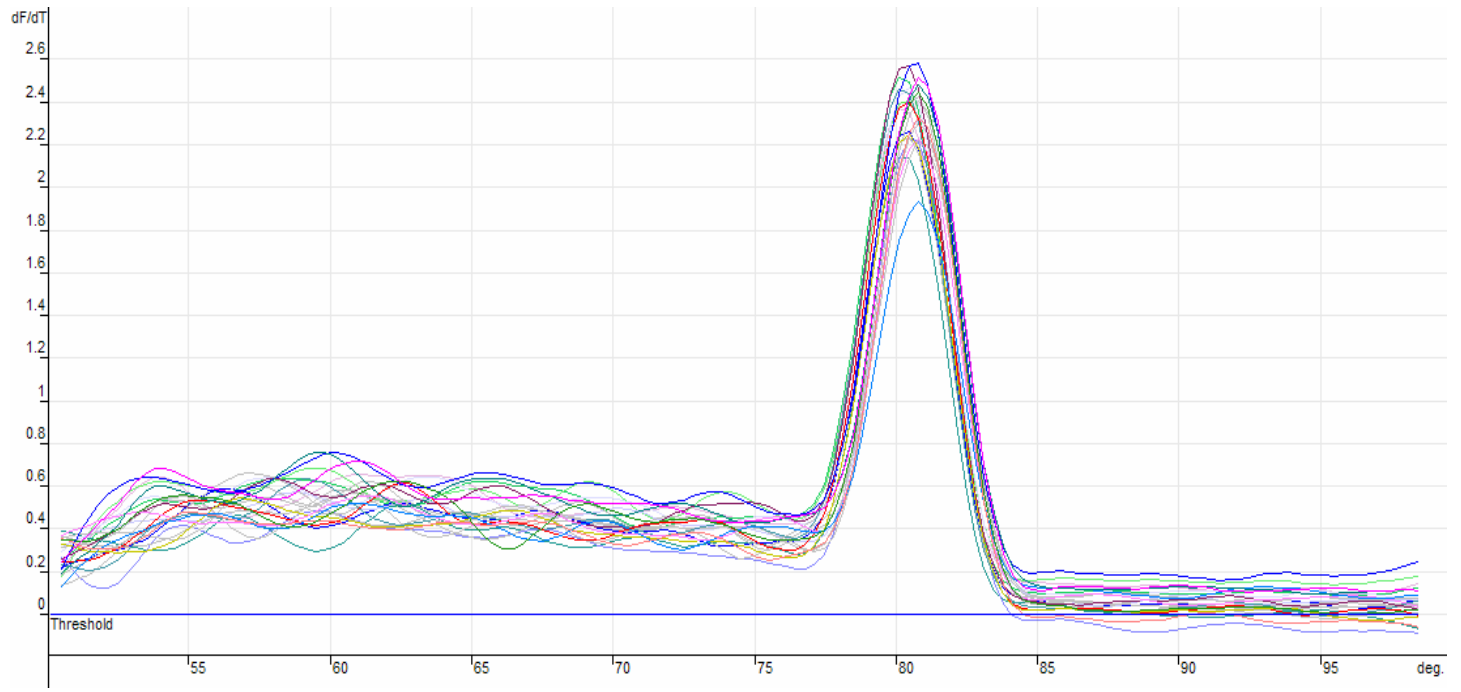

TBP

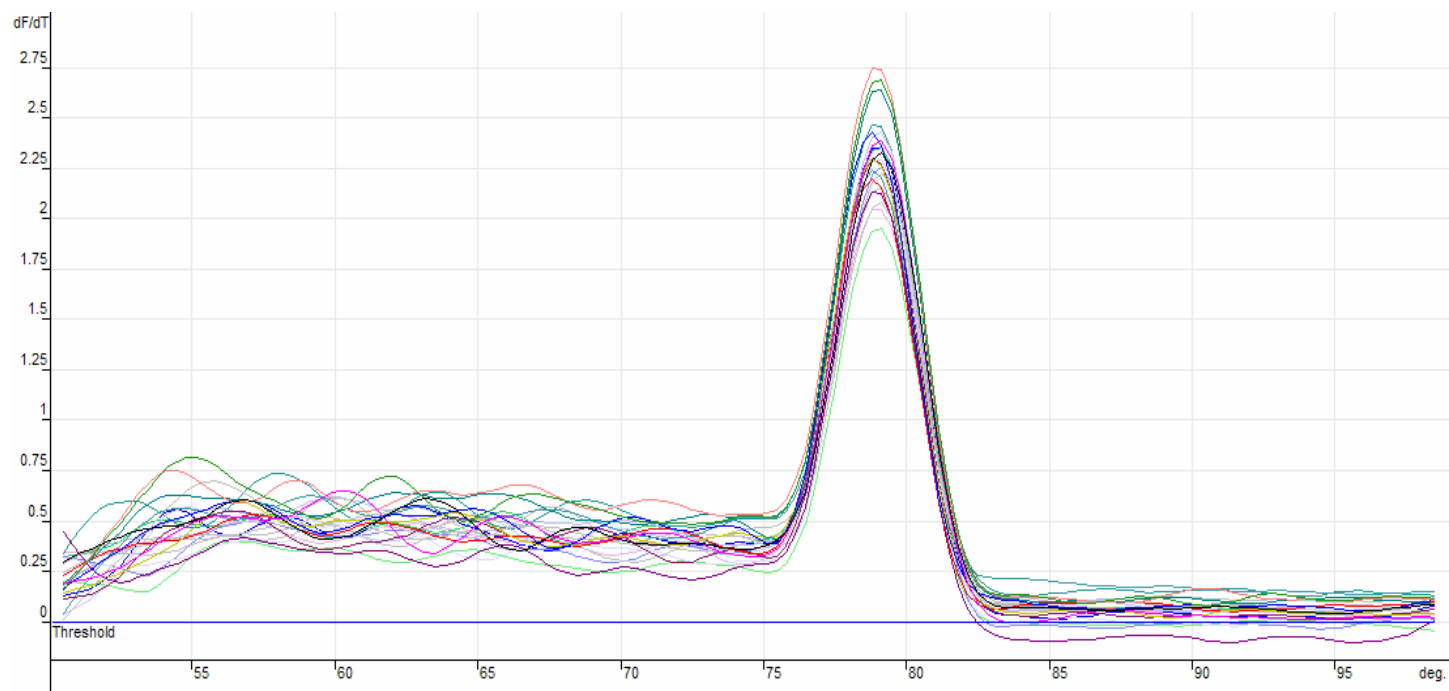

Supplement: Additional file 5 — Melting curve analysis. Melting curve analysis of 8 different primer sets for reference genes in humans. [file 1471-2199-9-78-S5.pdf]

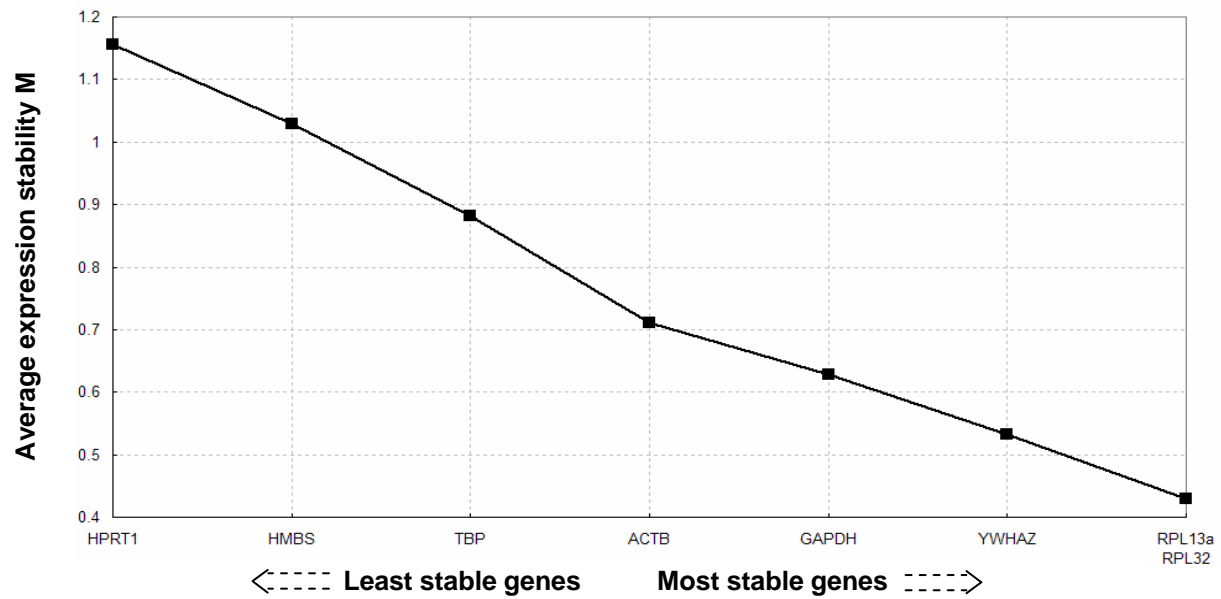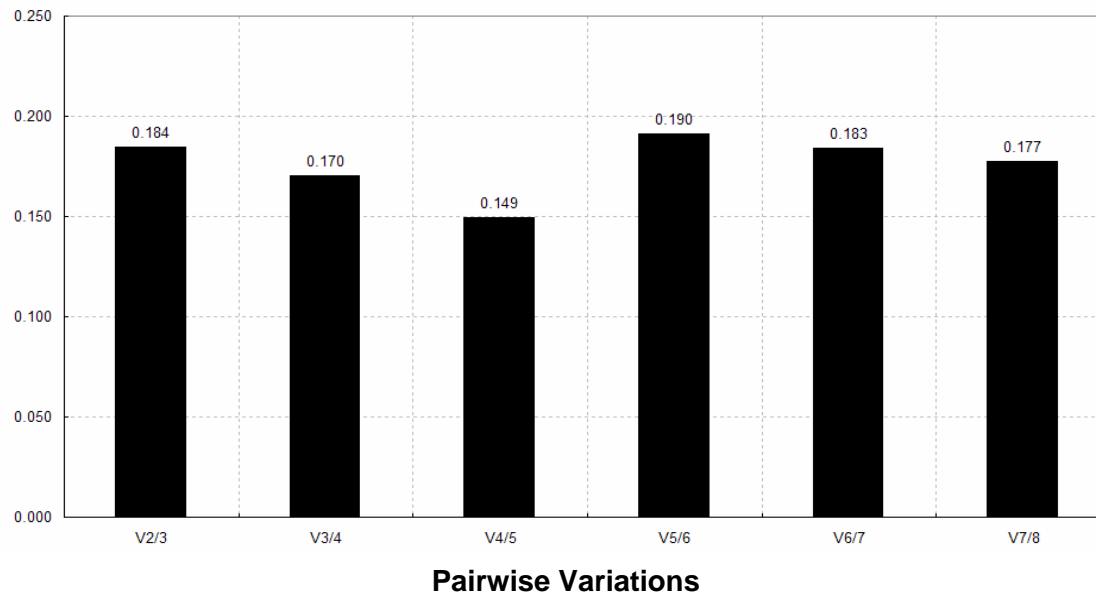

Supplement: Additional file 6 — Selection of the most suitable reference genes for normalization using geNorm analysis using the unknown rhesus monkey samples for validation. [file 1471-2199-9-78-S6.pdf]
